# Identification and Characterization of an Unusual Class I Myosin Involved in Vesicle Traffic in *Trypanosoma brucei*

**DOI:** 10.1371/journal.pone.0012282

**Published:** 2010-08-19

**Authors:** Diana Spitznagel, John F. O'Rourke, Neal Leddy, Orla Hanrahan, Derek P. Nolan

**Affiliations:** 1 Molecular Parasitology Group, School of Biochemistry and Immunology, Trinity College Dublin, Dublin, Ireland; 2 European Bioinformatics Institute, Wellcome Trust Genome Campus, Hinxton, United Kingdom; 3 Centre for Microscopy and Analysis, Trinity College Dublin, Dublin, Ireland; University of Massachusetts Amherst, United States of America

## Abstract

Myosins are a multimember family of motor proteins with diverse functions in eukaryotic cells. African trypanosomes possess only two candidate myosins and thus represent a useful system for functional analysis of these motors. One of these candidates is an unusual class I myosin (TbMyo1) that is expressed at similar levels but organized differently during the life cycle of *Trypanosoma brucei*. This myosin localizes to the polarized endocytic pathway in bloodstream forms of the parasite. This organization is actin dependent. Knock down of TbMyo1 results in a significant reduction in endocytic activity, a cessation in cell division and eventually cell death. A striking morphological feature in these cells is an enlargement of the flagellar pocket, which is consistent with an imbalance in traffic to and from the surface. In contrast TbMyo1 is distributed throughout procyclic forms of the tsetse vector and a loss of ∼90% of the protein has no obvious effects on growth or morphology. These results reveal a life cycle stage specific requirement for this myosin in essential endocytic traffic and represent the first description of the involvement of a motor protein in vesicle traffic in these parasites.

## Introduction

African trypanosomes are the causative agents of sleeping sickness in humans and trypanosomiasis in cattle. These parasites have significant human and veterinary health implications throughout sub Saharan Africa. Long slender forms of the mammalian bloodstream and procyclic forms of the tsetse fly vector are the main proliferative stages during the life cycle. Bloodstream forms are obligatorily dependent on the uptake of growth factors, such as transferrin and lipoproteins, from their mammalian hosts. This essential traffic is polarized and limited to a small invagination of the plasma membrane, termed the flagellar pocket, located at the posterior end of the cell. Endocytic activity is significantly higher in bloodstream forms where it may also have a defensive role in recycling of the variant surface glycoprotein (VSG) to allow clearance of surface bound antibodies [1], [2]. The endocytic pathway of *T. brucei* has been the focus of much attention during the past decade and significant progress has been made in understanding vesicle traffic at various levels [3], [4], [5], [6], [7], [8]. However, the physical mechanisms that underlie this traffic in trypanosomes have yet to be described. Nevertheless, there is clear evidence that actin is required [9], while myosins are known to be involved in vesicle traffic in other eukaryotes [10], [11]. These considerations prompted us to investigate myosin function in *T. brucei*.

Myosins are a conserved superfamily of ATPase mechanoenzymes that consist of an N-terminal motor domain, a neck and a C-terminal tail region. The first myosins to be described were muscle or “conventional” myosins followed by the unconventional myosins [12], [13]. Currently, a total of 24 different myosin classes have been described and the largest single group is the class I family [14]. These myosins are smaller than the conventional or class II myosins, are single headed and unable to self-associate into bipolar filaments. The tail region of class I myosins varies in length, sequence and domain structure and is thought to specify the function of these motors in numerous cellular processes [14], [15], [16], [17], [18], [19], [20], [21], [22], [23], [24], [25]. The precise function of many of these myosins remains to be determined and functional studies have been complicated by the presence of multiple myosin forms in most eukaryotes. The situation in African trypanosomes is less complex as genome sequencing has identified only two candidate myosin genes. This study characterizes one of these candidates, an atypical class I myosin, using a variety of approaches to investigate the expression, location and function of this motor.

## Results

### An unusual class I myosin in *T. brucei*

The annotated genome of *T. brucei* contains two putative myosin genes, Tb11.01.7990 and Tb927.4.3380, with respective UniProt entries of Q381F5 and Q585L2 (Table S1). The latter entry (Q585L2/Tb927.4.3380) corresponds to a protein of ∼130 kDa, which the NCBI database mapped to the GenBank accession number AAZ10929.1. A global Needleman-Wunsch alignment confirmed that both entries were identical. A previous phylogenetic classification, based on distance matrix-based analysis of myosin head domains (using protdist/neighbor programs in the Phylip package), concluded that this protein was a class I myosin [14]. We performed an additional, complimentary analysis of the protein to confirm this classification and to investigate further the domain structure of the protein. A character-based maximum likelihood phylogenetic analysis of 235 myosins from a variety of taxonomic divisions revealed that Q585L2 clustered with class I myosins using three different alignment approaches (Figs. S1, S2, S3). This class I designation was further supported by analysis of the scores/e-values associated with matches of 37 separate PTHR13140 subfamily-models to Q575L2 and the other myosins (run locally using HMMER). The highest matching sub-model was SF31, mirroring that of other class I myosins (not shown). On the basis of these results we adopted the name TbMyo1 for *T. brucei* myosin class I as proposed by Foth *et al*. [14].

**Figure 1:**
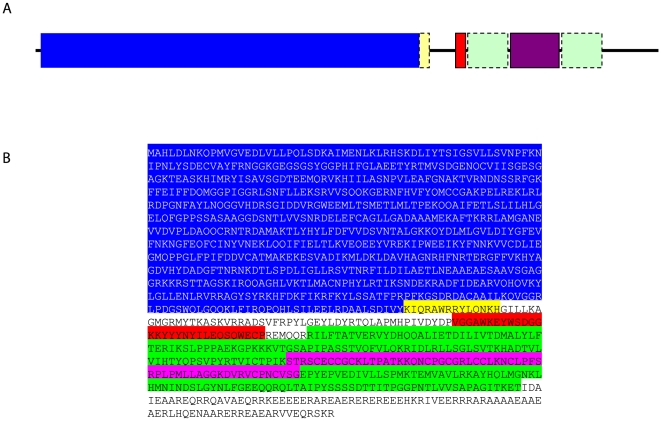
Domain structure of TbMyo1. Panel A. A model of the domain structure of TbMyo1 was constructed using data from InterPro matches (Table S2) and alignments of the head (Fig. S4) and tail (Fig. S5) regions. The model presents the following domain structure: blue, myosin head (IRP001609); yellow, IQ motif (degenerate); red, WW domain (IPR001202); green, TH1 domain (IPR0010926); purple, FVYE domain (IPR0011011, IPR017455. Panel B. The primary structure of TbMyo1 with the relevant domains indicated by the colours utilized in panel A.

The domain structure of TbMyo1 was investigated by searching for matches with known signatures (Table S2). There were multiple matches within the N-terminal region (∼775 residues) that mapped to the myosin head, motor domain InterPro Entry IPR001609. Although other signature hits did not map to an InterPro Entry, they were associated with the myosin head domain. An alignment of the N-terminal motor domain of TbMyo1 with other class I myosins that segregated into the same phylogenic group (see Fig. S1) revealed the presence of conserved ATP and actin binding regions plus a single IQ motif. Interestingly, the latter motif did not show up during the signature analysis (Fig. S4). Class I myosins are often referred to as short or long tailed forms depending on their tail region which can vary considerably in length, sequence and presence of tail homology domains (TH) present [15]. All class I myosins described to date appear to contain a TH1 domain rich in basic residues. This domain is thought to be involved in membrane interactions through association with acidic phospholipids but without apparent specificity to the acidic head group. The tail of class I myosins may also contain additional domains, such as a TH2 domain rich in glycine, proline and either alanine or glutamine, which in some cases has been shown to be capable of ATP-independent binding of actin, an SH3/TH3 domain or in fungal isoforms a C-terminal acidic domain [26], [27], [28], [29], [30], [31]. A comparative analysis of the tail region of TbMyo1 revealed a number of interesting features (Fig. S5 and Table S2). First, the TbMyo1 tail commences with a WW domain (IPR001202), a domain associated with protein-protein interactions [32] but not usually found in class I myosins. This feature appears to be restricted to myosins from kinetoplastids and possibly choanoflagellates (not shown). Second, although a TH1 domain is present, the first 18 residues appear to be missing and the domain itself is disrupted by the insertion of a putative zinc-binding FYVE domain (IPR011011 and IPR017455). This insertion occurs immediately after a highly conserved lysine located roughly in the middle of the TH1 domain (Fig. S5 and Table S2). The remaining C-terminal region of the TH1 domain follows the FYVE domain and the tail ends with a region rich in acidic and basic residues that differs from the acidic region of fugal isoforms (Fig. S5). Finally, TbMyo1 lacks the TH2 and SH3 domains found in long tailed class I myosins. Thus, the domain structure of TbMyo1 indicates that it is an unusual member of the short tailed class I group of myosins (Fig. 1).

Analysis of the genomes of other kinetoplastids, *e.g. T. cruzi* and *Leishmania*, revealed the presence of a similar class I myosin that also contained a putative FYVE domain in the tail region [14]. A series of HMMer searches were performed using various FYVE domain models against a set of 1,700 myosins from the UniProt database to determine whether this feature was kinetoplastid specific (Tables S3 and S4). This analysis revealed that myosins containing a FYVE domain are present only in kinetoplastid genomes and possibly the marine diatom *Phaeodactylum tricornutum* (C6JVZ1). The significance of the FYVE domain in TbMyo1 was assessed within the context of other FYVE-domain containing proteins in *T. brucei* by another HMMer analysis performed using various FYVE domain models (Tables S5). Out of a total of 19 *T. brucei* proteins with definite hits to the FYVE-domain, TbMyo1 (Q585L2) was ranked at number 18 (Table S5). While this ranking appears low, the FYVE domain of kinetoplastid myosins display relatively similar ranks, scores and e-values to those of known FYVE-domain containing proteins (Table S6). Interestingly, FYVE domains have the potential to target proteins to membranes via interaction with phosphatidylinositol-3-phosphate [33] and in TbMyo1 this domain disrupts another domain (TH1) that is also involved in membrane binding [26], [27], [28], [29].

### Expression of TbMyo1

**Figure 2:**
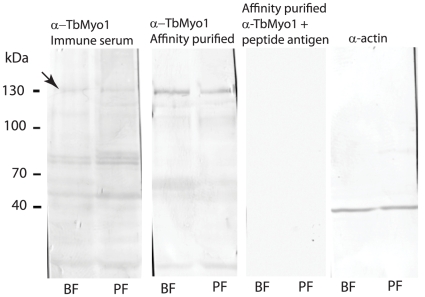
Western Blot analysis of TbMyo1 protein in *T. brucei* bloodstream and procyclic form. Total cell lysates of bloodstream (BF) and procyclic forms (PF) were subjected to a western blot analysis using immune serum or affinity purified antibodies raised against a peptide specific to TbMyo1. A protein band was detected in both life forms that migrated at ∼130 kDa (arrow). Detection of this protein was significantly enhanced using the affinity purified antibodies. The specificity of the affinity purified antibodies was demonstrated by including the peptide antigen (10 µg.ml^−1^) with the primary antibody. A loading control was performed using antibodies against trypanosome actin [9]. A total of 5×10^6^ cells were loaded per lane.

The relative level of expression of the *TbMyo1* transcript was investigated using qRT-PCR and relative expression ratios (bloodstream/procyclic form) of 2.8±0.3 and 1.7±0.5 (both mean ± SD of three determinations) were obtained using total RNA and purified mRNA fractions respectively. Polyclonal antibodies were raised in rabbits against a TbMyo1 specific peptide and used to investigate expression of TbMyo1 at the protein level (Fig. 2). A protein that migrated with the expected size of TbMyo1 (∼130 kDa) was detected in bloodstream and procyclic forms using the immune serum. Detection of this protein was significantly enhanced following affinity purification of the immune serum. These antibodies were specific for TbMyo1 as this protein was not detected using preimmune serum (not shown) nor when the peptide antigen was included with the purified primary antibody (Fig. 2). A comparison with the actin loading control indicated similar levels of expression of TbMyo1 in both life cycle forms.

### Location of TbMyo1

**Figure 3:**
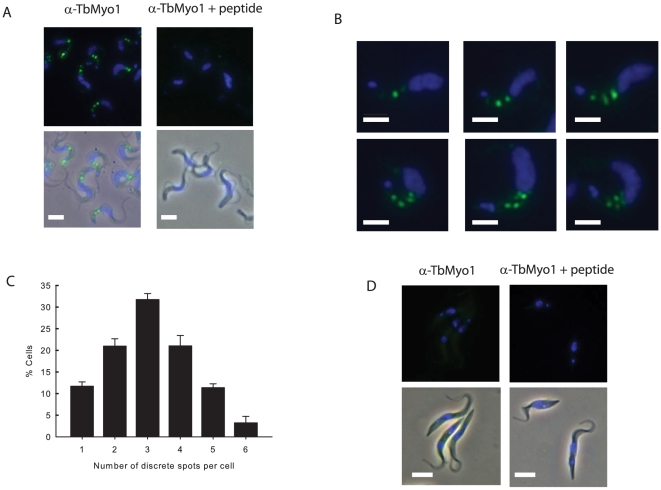
Localization of TbMyo1 in bloodstream and procyclic forms of *T. brucei*. Bloodstream forms were purified from infected blood, fixed and then processed for immunofluorescence using affinity purified anti-TbMyo1 antibodies as described in the methods. The cells were visualized using a Zeiss Axiovert 100 fluorescence microscope. The images were captured and processed using Axiovision software. Bar  = 5 ųm. Panel A. The upper panels present the fluorescence image and reveal the location of TbMyo1 (green) and the nucleus/kinetoplast (blue). The lower panels present the corresponding merge with the phase contrast view. Nonspecific binding was investigated by including the peptide antigen (10 µg.ml^−1^) along with the α-TbMyo1 affinity purified antibodies. Panel B. A series of images from the same population of bloodstream forms demonstrated that TbMyo1 was present in discrete spots that varied in number, signal intensity and relative location between the nucleus and kinetoplast. Panel C. Analysis of the number of TbMyo I spots in cells. Three different fields of 100 cells were analysed and the results were expressed as mean ± SEM. The analysis revealed a distribution of TbMyo1 spots ranging from 1 to 6 spots per cell. Panel D. Cultured procyclic forms were fixed and processed for immunofluorescence as described in the methods. The upper panels present the fluorescence image and reveal a weak, uniform signal distributed throughout the cell. The lower panels present the fluorescence merged with the corresponding phase image. Nonspecific binding was investigated by including the peptide antigen (10 µg.ml^−1^) along with the α-TbMyo1 affinity purified antibodies.

**Figure 4:**
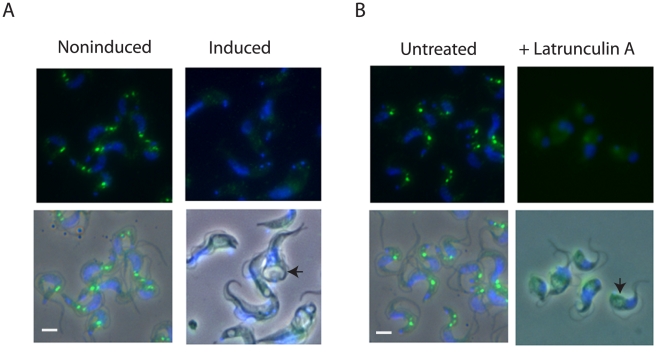
The polarized location of TbMyo1 in bloodstream forms depends on the actin cytoskeleton. The images are presented as the fluorescence image (upper) and the corresponding phase merge (lower). The enlarged flagellar pocket is visible as a phase light vacuole (arrow) located in the posterior region of the induced RNAi cell line or in latrunculin-treated cells. Bar  = 5 ųm. Panel A. The location of TbMyo1 (green) in induced (30 h) and noninduced conditional actin RNAi cells. Panel B. The location of TbMyo1 (green) in bloodstream forms incubated in the absence or presence of Latrunculin A (200 nM) for 4 h at 37°C.

The location of TbMyo1 was investigated in fixed bloodstream and procyclic cells using the affinity purified antibodies (Fig. 3). These studies demonstrated that TbMyo1 was located as a series of discrete spots that were distributed exclusively between the kinetoplast and nucleus in bloodstream forms (Fig. 3A). These spots were specific for TbMyo1 since they were not detected when the peptide antigen was included along with the primary antibody. While the precise number and intensity of TbMyo1 spots varied from cell to cell, they were always located in the posterior region of the cell associated with vesicle traffic (Fig. 3B). An analysis of several populations of cells revealed that the number of TbMyo1 positive spots varied and ranged from one to as many as six spots per cell that differ in their intensity and relative location between the kinetoplast and nucleus (Fig. 3C). This variable distribution suggested that there was a dynamic aspect to the polarized location of TbMyo1 in populations of bloodstream forms. In contrast the protein appeared to be distributed throughout procyclic forms as indicated by the weak uniform cellular fluorescence observed (Fig. 3D). This weak signal was also specific for TbMyo1, as it was not detected when the peptide antigen was included with the primary antibody. These results demonstrated a differential organization of TbMyo1 in bloodstream and procyclic forms that was similar to that previously observed for actin [9]. Therefore, the effect of disruption of the actin cytoskeleton on the location of TbMyo1 was investigated (Fig. 4). Repression of actin expression using RNAi clearly affected the distribution of TbMyo1 (Fig. 4A). Under noninduced conditions TbMyo1 was located in a polarized manner in the posterior region of the cell. However, the distribution was more diffuse and less polarized in the induced cells. The presence of an enlarged flagellar pocket was consistent with the loss of actin in these cells as observed previously [9]. Disruption of the actin cytoskeleton using latrunculin A, a membrane permeable agent that binds specifically to monomeric G-actin and disrupts filamentous actin in cells [34], [35], also resulted in a more diffuse distribution of TbMyo1 (Fig. 4B). Neither of these treatments had an obvious effect on the uniform distribution of TbMyo1 in procyclic cells (data not shown). These data demonstrated that the polarized, spot-like distribution of TbMyo1 in bloodstream forms was dependent on actin, while the effect of latrunculin was consistent with an interaction with filamentous actin. However, direct colocalization studies with actin were hampered by the fact that both primary antibodies were from the same host species. Attempts at localization experiments using biotin labelled anti-TbMyo1 antibodies were unsuccessful, possibly because biotinylation affected recognition of the antigen. The use of commercial mouse monoclonal antibodies against actin (β-actin C4 from Santa Cruz) was also unsuccessful as these antibodies failed to detect trypanosomal actin either in blotting or immunofluorescence experiments (not shown). As a compromise we compared the location of TbMyo1 and actin in separate localization experiments using the same cells and observed a broadly similar distribution of both proteins between the kinetoplast and nucleus consistent with their joint presence throughout the endocytic pathway (Fig. S6).

**Figure 5:**
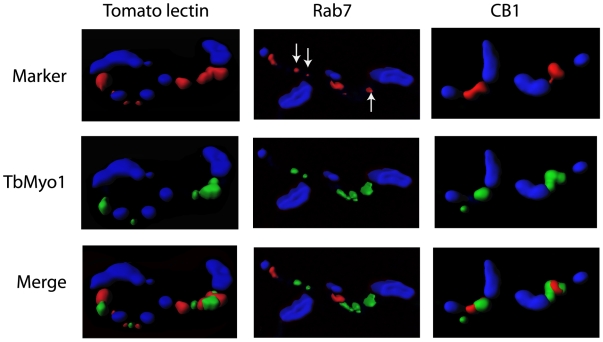
Colocalization of TbMyo1 with elements of the endocytic pathway in bloodstream forms. Purified *T. brucei* bloodstream forms were processed for immunofluorescence as described in the methods section. The cells were probed with affinity purified TbMyo1 antibody (green) and colocalizations were performed with Texas red labelled tomato lectin, rat anti-Rab 7 or mouse anti-CB1 (red). The cells were incubated with lectin for 1 min at 37°C before being diluted 10-fold into the ice-cold buffer, washed and then processed for immunofluorescence. The anti-Rab7 and anti-CB1 antibodies were detected using the relevant Alexa 568 labelled secondary antibody (red). In all cases the position of the nucleus and kinetoplast was revealed by DAPI staining (blue). The cells were examined using an Olympus Fluoview 1000 confocal microscope. The images were captured using an “UPLSAPO 60X O NA:1.35” objective, optically sectioned at 0.35 ųm/slice and the combined Z-stack images were reconstructed and iso-surface rendered using Imaris (v6.4.2) imaging software. The upper panels present the location of the markers, the middle panels represent the location of TbMyo1 and the lower panels represent the merge of both signals. Arrows indicate small Rab7 compartments where there appeared to be full overlap with TbMyo1 signal.

Endocytic traffic in bloodstream forms is highly polarized and concentrated within the posterior region of the cell. The possible colocalization of TbMyo1 with the flagellar pocket, endosomal and lysosomal compartments was investigated by confocal microscopy (Fig. 5). We employed the uptake of tomato lectin at 37°C for 1 min as a marker for the flagellar pocket and early endosomes [36], Rab7 as a marker for the late endosomal compartment [37], [38] and the CB1 epitope of the lysosomal membrane glycoprotein p67 as a marker of the lysosomal compartment [39], [40]. There was some degree of colocalization of TbMyo1 with all of these compartments but a full overlap of the two signals was not observed with the possible exception of some of the smaller Rab7 positive compartments (see arrows Fig. 5). A quantitative analysis of the extent of colocation in these cells was performed on the original Z-stack data prior to iso-surface rendering using the “Coloc” module (Bitplane). This analysis predicted a Pearson's correlation coefficient of 0.47, 0.46 and 0.53 for colocalization of TbMyo1 with tomato lectin, Rab7 and CB1 respectively. These values are consistent with a signal overlap of TbMyo1 with each marker of approximately 50% within the population. To assess possible interaction with cargo-containing vesicles undergoing traffic additional colocalization studies were performed using tomato lectin as a surrogate cargo as described previously [41]. Again these studies revealed partial colocalization of TbMyo1 with the internalised tomato lectin after 5 or 10 min incubation at 37°C. However, there was no overlap with TbMyo1 when the cells were incubated on ice where the lectin remained associated with a single spot located close to the kinetoplast consistent with location in the flagellar pocket (Fig. S7).

### TbMyo1 is essential in bloodstream forms of *T. brucei*

**Figure 6:**
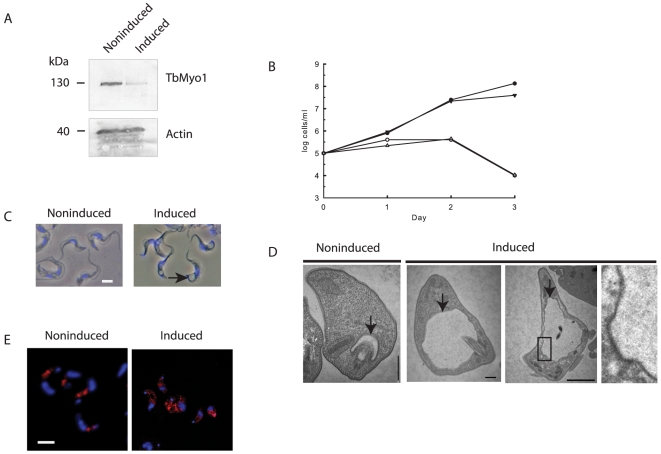
The effect of knock down of TbMyo1 in bloodstream forms o*f T. brucei.* Panel A. Western Blot analysis of TbMyo1 expression in a knock down cell line cultured for 24 h in the absence (noninduced) or presence of tetracycline (induced). Decreased expression of the TbMyo1 130 kDa protein is clearly visible in the induced cells. Each lane contained 5×10^6^ cells and a loading control was performed with antibodies against trypanosome actin. The loss of TbMyo1 had no effect on expression of actin. Panel B. The effect of knock down of TbMyo1 on the growth of bloodstream form RNAi cells. Two different bloodstream RNAi clones were grown in the presence (○, ▵) or absence of tetracycline (•, ▾). Panel C. The effect of the knock down of TbMyo1 on cellular morphology. The cells were cultured in absence (noninduced) or presence (induced) of tetracycline for 24 h, fixed and examined by phase contrast microscopy. A large phase-light vesicle or vacuole (arrow) located between the kinetoplast and nucleus (blue) was visible in the induced cells. Bar  = 5 ųm. Panel D. Ultrastructural analysis of the effect of knock down of TbMyo1. The TbMyo1 RNAi bloodstream cells were cultured in the absence (noninduced) or presence (induced) of tetracycline for 24 h. The cells were processed for electron microscopy as described in the methods section. Sections through the flagellar pocket of a noninduced control cell and induced cells are shown. Enlargement of the flagellar pocket (arrow) is clearly visible in the induced cells. A noticeable feature of the enlarged pocket in TbMyo1 RNAi cells was the presence of the inward deformations of the flagellar pocket membrane but an absence of vesicles forming from the pocket. One of these regions (boxed) is shown at higher magnification. Bar  = 500 nm. Panel E. Knock down of TbMyo1 affects the distribution of clathrin in cells. Bloodstream TbMyo1 RNAi cells were cultured in the absence (noninduced) or presence (induced) of tetracycline for 24 h. The cells were fixed and immunolocalizations were performed using an anti-clathrin antibody (red). The kinetoplast and nucleus are also shown (blue). Bar  = 5 ųm.

The functional role of TbMyo1 was assessed by conditional RNAi using a construct that allowed the tetracycline-inducible expression of a double stranded RNA corresponding to 745 nucleotides unique to the tail region of TbMyo1. Induction of the dsRNA resulted in a 50% decrease in the *TbMyo1* transcript within 24 h as indicated by the relative expression ratio (induced/noninduced) of 0.5±0.1 determined by qRT-PCR (mean ± SD of three separate determinations). A western blot analysis confirmed this loss at the protein level and revealed a relative reduction of ∼5-fold in TbMyo1 in the induced cells (Fig. 6A). Knock down of TbMyo1 clearly had a deleterious effect on the growth of bloodstream forms as cell division ceased shortly after induction of the dsRNA and cell death occurred about 2–3 days later (Fig. 6B). Analysis of the kinetoplast/nucleus ratio indicated that the effect on growth was primarily at the level of cytokinesis as there was a progressive increase in the number of multinucleated cells in the population following induction of the dsRNA (data not shown). During this period cellular morphology changed dramatically as cells became increasingly enlarged and distorted at the posterior end of the cell. Examination of these cells using phase contrast microscopy revealed the presence of a phase light vesicle/vacuole in this region of the cell (Fig. 6C). Ultra structural analysis of the cells demonstrated that this vesicle represented a grossly enlarged flagellar pocket (Fig. 6D). Significantly, other structures such as the nucleus or flagellum were not affected by loss of TbMyo1. This morphological phenotype was consistent with a decrease in endocytic activity but continuous membrane traffic to the surface as observed previously in clathrin or actin RNAi cell lines [4], [9]. A noticeable feature in many sections of the enlarged pocket in TbMyo1 RNAi cells was the presence of large inward deformations of the flagellar pocket membrane but an absence of vesicles pinching off from the enlarged pocket. The loss of TbMyo1 also had a disruptive effect on the distribution of clathrin, which became less polarized, more diffused and scattered (Fig. 6E). This disruptive effect on clathrin distribution was also consistent with a dysfunction in the formation and traffic of coated vesicles.

**Figure 7:**
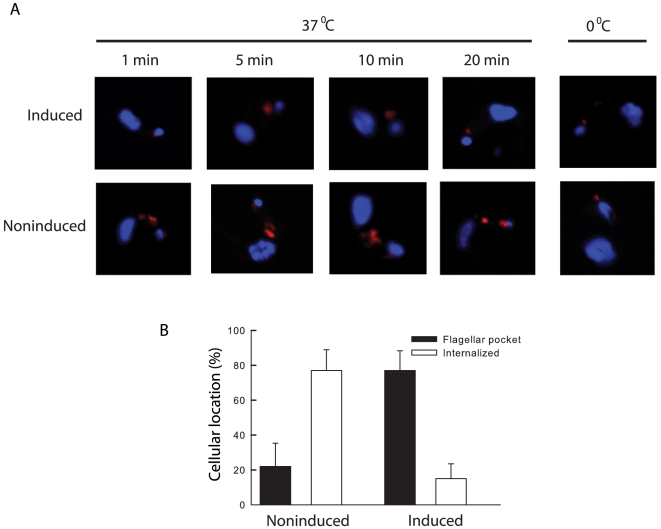
Knock down of TbMyo1 inhibits endocytosis in bloodstream forms of *T. brucei.* Bloodstream TbMyo1 RNAi cells were cultured in the absence (noninduced) or presence (induced) of tetracycline for 24 h. Endocytic activity was assessed microscopically by observing the internalisation of Texas red labelled tomato lectin. Panel A. The uptake of tomato lectin (red) into cells incubated at 37°C for various times. Typically only a single spot located close to the kinetoplast (small blue spot) was visible in the induced cells. This location was identical to that observed when the experiment was performed at 0°C. There were additional stronger signals closer to the nucleus (larger blue spot) in noninduced cells consistent with entry of the lectin into intracellular compartments. Panel B. Histograms show quantification of the location of labelled tomato lectin in induced and noninduced TbMyo1 RNAi cells after an incubation of 20 min. Cells containing a single spot of fluorescence located close to the kinetoplast, e.g. as observed at 0°C, were scored as having a flagellar pocket location. Cells with clear, additional labelling located closer to the nucleus were scored as having internalised the lectin. A total of 100 cells were scored in this way and the data represent the mean ± SEM from three different RNAi experiments.

The role of TbMyo1 in endocytosis was investigated directly by a microscopic analysis of the uptake of Texas-red labelled tomato lectin (Fig. 7). Internalization of the lectin was clearly inhibited when TbMyo1 expression was repressed. Even after an incubation of 20 min at 37°C, the lectin remained associated with a single spot located close to the kinetoplast in many cells, consistent with an exclusively flagellar pocket location (Fig. 7A). Indeed this location was identical to that observed when the experiment was performed at 0°C, *i.e.* under conditions where there is no endocytic traffic. In contrast even after 1 min there was obvious uptake of the lectin in the noninduced cells as judged by the appearance of additional fluorescence labelling of regions located closer to the nucleus, consistent with the presence of the lectin in endosomal/lysosomal compartments. A quantitative analysis of several populations of these cells demonstrated a 4-fold decrease in the amount of lectin internalized and a corresponding increase in the number of cells where only flagellar pocket binding was observed (Fig. 7B). Taken together, these data demonstrated that TbMyo1 plays an essential role in endocytic traffic in bloodstream forms and provided the first unequivocal demonstration of the involvement of a motor protein in this traffic in these parasites.

**Figure 8:**
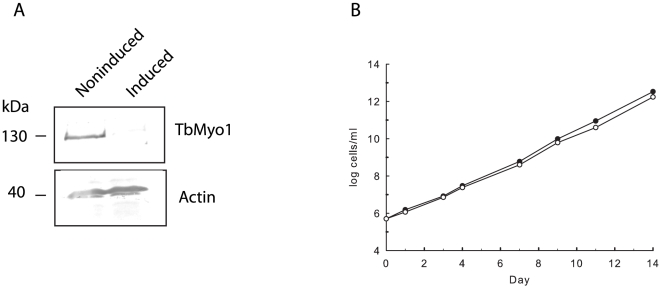
Knock down of TbMyo1 in procyclic forms of *T. brucei*. Panel A. Procyclic form TbMyo1 RNAi cells were grown in the absence (noninduced) or presence (induced) of tetracycline for 24 h. A western blot analysis of total cell lysates demonstrated a significant loss of TbMyo1 in the induced cells. A total of 5×10^6^ cells were loaded in each lane. A loading control was performed on the same blot with antibodies against trypanosome actin. Panel B. The effect of the loss of TbMyo1 in procyclic RNAi cells cultured in the presence (○) or absence (•) of tetracycline.

The functional role of TbMyo1 was also investigated in procyclic form RNAi cells. Induction of the dsRNA resulted in a 90% loss of the *TbMyo1* transcript within 24 h as indicated by the relative expression ratio (induced/noninduced) of 0.11±0.06 determined by qRT-PCR (mean ± SD of three separate determinations). A western blot analysis demonstrated that TbMyo1 was barely detectable in the induced cells (Fig. 8A). However, this large loss of TbMyo1 had no effect on cellular growth as both induced and noninduced cells grew at approximately the same rate with a doubling time of ∼15 h (Fig. 8B). Furthermore, no obvious motility defects or morphological aberrations were observed in the induced cells using either phase contrast or transmission electron microscopy (not shown). Therefore, even though TbMyo1 expression is similar in bloodstream and procyclic forms, at least 90% of the protein appears to be dispensable in the latter cells.

## Discussion

African trypanosomes possess two putative myosins, one of which, TbMyo1, corresponds to an atypical short tailed class I myosin. The tail region of TbMyo1 is unusual as it contains domains not normally associated with myosins, i.e. WW and FYVE, and also appears to lack a functional TH1 domain. What might be the significance of these alterations to the tail structure? It is striking that the TH1 domain is disrupted by insertion of the FVYE domain. The former domain is found in all class I myosins and is thought to be involved in membrane binding through association with acidic phospholipids but without any apparent specificity with respect to the acidic head group [26], [27], [29]. In contrast, FYVE domains specifically target proteins to membranes containing phosphatidylinositol–3-phosphate (PtdIns(3)P) [33], [42]. Thus, replacing the TH1 with a FYVE domain has the potential to alter the target specificity of TbMyo1 from membranes rich in acidic phospholipids to those rich in PtdIns(3)P. These membranes may include those of the early endosomes and related compartments where PtdIns(3)P is produced by class III PI 3-kinases [43]. Significantly, FYVE domain containing proteins are known to be involved in vesicle traffic in other eukaryotes [42], while knock down of the single PI 3-kinase in *T. brucei* had a deleterious effect on vesicle traffic [44]. These considerations support the view that PtdIns(3)P and FYVE domain containing proteins, such as TbMyo1, are likely to be involved in vesicle traffic in trypanosomes.

The results presented here demonstrated that TbMyo1 plays an important role in traffic in bloodstream forms. First, TbMyo1 was located exclusively within the region of the cell associated with endocytic activity. A distinctive feature was the location of TbMyo1 into discrete spots, which varied in number and intensity from cell to cell. These spots colocalized, at least partially, with various elements of the endocytic pathway as well as compartments containing internalized cargo, suggesting that TbMyo1 may be involved in traffic throughout the endocytic pathway from the flagellar pocket via endosomes to the lysosomal compartment. The variation in TbMyo1 distribution in terms of the number, intensity and relative location of discrete spots between kinetoplastid and nucleus probably reflects variation in the levels of endocytic activity and vesicle trafficking events in individual cells within the population. Second, knock down of TbMyo1 resulted in a lethal defect in vesicle traffic in bloodstream forms. There was a significant decrease in traffic from the flagellar pocket as demonstrated by a significant reduction (∼80%) in the uptake of tomato lectin in the induced cells. This reduction in endocytic activity was comparable to that previously observed when clathrin or actin expression was repressed [4], [9]. Endocytic traffic is exclusively clathrin-mediated in *T. brucei* [4] so it was not surprising that the loss of TbMyo1 also affected the distribution of clathrin. Perhaps the most dramatic evidence for a role in endocytic traffic was the enlargement of the flagellar pocket, which was a consistent feature of the TbMyo1 knock down cells. This defect, which has been termed “big-eye”, also occurs in clathrin and actin RNAi cell lines where it has been shown to result from an imbalance in traffic that occurs when endocytic activity is reduced but traffic of newly synthesized proteins and membrane to the surface continues [45]. The phenotypic link between loss of clathrin, actin and TbMyo1 is striking and is clearly related to their collective requirement during the initial steps of endocytosis in trypanosomes.

Interestingly, the enlarged flagellar pocket in the TbMyo1 RNAi cells was characterized by obvious, large deformations (>50 nm) of the pocket membrane into the cytoplasm and at times there appeared to be a thickening of the cytoplasmic face of these deformations (Fig. 5D). These deformations were not observed in actin or clathrin RNAi lines where the membrane of the enlarged pocket had a uniform smooth appearance [4], [9]. This differential effect on the membrane of the flagellar pocket suggests that clathrin and actin might be required prior to TbMyo1 during the initial formation of membrane invaginations in trypanosomes but that TbMyo1 is involved at subsequent steps such as vesicle scission and movement into the cytoplasm. Although these deformations are somewhat larger and shaped differently to the initial tubular invaginations of the plasma membrane of yeast [46], this temporal sequence is similar to current models for actin dependent endocytosis. In these models actin assembly around the clathrin coat of the nascent vesicle precedes recruitment of the myosin motor Myo5, which drives subsequent elongation of the invagination and cooperates with amphiphysins in the fission event [47], [48]. In higher eukaryotes the transition from clathrin coated pits to closed endocytic vesicles is thought to involve multiple interactions between the actin cytoskeleton, syndapins and dynamins, which are involved in the pinching off process [49]. The trypanosome genome lacks syndapin and amphiphysin candidates, while the single dynamin is apparently not required for endocytosis [50]. Therefore, it is possible that TbMyo1 may be directly involved in vesicle invagination and scission from the flagellar pocket membrane. Interestingly, loss of myosin IB in *Dictyostelium* [51], [52], which is closely related to TbMyo1, resulted in an accumulation of small vesicles clustered around endosomes to which they appeared to be connected by a tether. Consequently, it has also been suggested that this myosin acts as a ‘pinch-ase’ to drive vesicle fission in *Dictyostelium*. A similar situation was observed in Myo5 yeast mutants which were characterized by a build up of invaginations of the plasma membrane [53]. However, the role of TbMyo1 may not be limited solely to events at the membrane of the pocket but probably extends to other steps in the endocytic pathway. This view fits with the distribution of TbMyo1 into discrete spots throughout the pathway in *T. brucei* and the extensive literature on the involvement of class I myosins at a variety of stages in vesicle transport, trafficking, endocytosis and membrane recycling in other cells [48], [54], [55], [56], [57]. The novel aspect of *T. brucei* is that TbMyo1 appears to be the sole myosin involved in this traffic. The other candidate myosin (Tb11.01.7990) is a member of a novel class XXI family [14] and the related protein in *Leishmania* is associated with the flagellum [58]. Moreover, we observed that transcripts for this myosin were not affected in the TbMyo1 knock down cells, while preliminary conditional RNAi experiments have revealed that loss of this other myosin had no obvious effect on vesicle traffic or growth of bloodstream forms (data not shown).

A number of insights follow from the finding that TbMyo1 is required for vesicle traffic in trypanosomes. First, it seems reasonable that the related class I myosins in other kinetoplastids, which also possess a FYVE domain [14], are similarly involved in vesicle traffic. This function is very different to the only other established role for myosin in protozoan parasites, which is gliding-based motility in apicomplexa [59], [60], [61]. Second, trypanosomes are extremely slender cells and vesicle traffic is extremely rapid, highly polarized and spatially restricted. The various compartments of the endocytic pathway are probably separated by no more than 1–2 µm in slender bloodstream forms [40], [62]. Given their proximity it might seem reasonable to question whether there was a role for motors in movement between these compartments. However, this is clearly the case in bloodstream forms. Thus, a motor is required even for movement between compartments in very close proximity. Third, these results have shown the usefulness of trypanosomes for investigating the role of myosin in vesicle traffic. Most eukaryotes, even other unicellular organisms, possess multiple forms and classes of myosins and consequently loss of any particular myosin may not reveal a phenotype owing to functional compensation by other myosins. Finally, the polymeric status of actin in trypanosomes has remained controversial. Direct evidence for the presence of filamentous or F-actin is lacking since no obvious microfilament structures have been observed in electron micrographs. In addition, universal F-actin binding agents such as the phallotoxins do not bind to actin from trypanosomes or indeed other protozoan parasites [63]. The essential requirement of a myosin in endocytic traffic in trypanosomes clearly implies an involvement of F-actin since there is no evidence that myosins interact with monomeric or G-actin *in vivo*. This conclusion is also supported by the effects of treatments known to affect the polymerization status of actin, *e.g.* repression of actin expression or treatment with latrunculin. The most likely reason for the failure to observe microfilament structures in *T. brucei* may be that they are short and dynamic as appears to the case in other protozoan parasites [64].

The function of TbMto1 in procyclic forms remains uncertain. Although expressed at similar levels in both life cycle forms, there was no obvious effect on growth or morphology of procyclic forms whenTbMyo1 levels were reduced to 10% of control levels (Fig. 7). While these results do not prove that TbMyo1 is dispensable in procyclic forms, there is clearly a significant level of redundancy. This feature may reflect the significantly reduced level of endocytic activity in these cells. However, it is interesting that expression of TbMyo1 is not down regulated as is the case for other proteins known to be essential for this traffic, such as clathrin and Rab11 [7]. Another striking feature is the differential organization of TbMyo1 in the two forms, which is also true for actin [9]. The differential functional requirement for actin andTbMyo1 during the life cycle of *T. brucei* might be connected to their spatial organization in bloodstream and procyclic forms. It is tempting to speculate that this feature is related directly to the levels of F-actin in the two forms. Perhaps in bloodstream forms actin is organised mainly into short dynamic filaments and associated with the endocytic pathway, whereas in procyclic forms the protein is present mainly as monomeric actin distributed throughout the cell. This view would be consistent with the observed differential sensitivity of both forms to latrunculin A [9]. In addition, we have preliminary data from experiments using Alexa labelled DNAse I, which binds specifically to G-actin, which indicates that G-actin is the main form of actin in procyclic forms. The distribution and requirement for TbMyo1 in both forms would follow directly from this arrangement of the actin cytoskeleton. This proposal raises the question of what mechanisms are involved in regulating actin filament formation and location in *T. brucei*?

## Methods

### Bioinformatics

Myosin sequences were obtained from GeneDB database (http://www.genedb.org). Entries for UniProt proteins were obtained from UniProtKB release 15.4. Blast searches were performed using NCBI stand-alone blast binaries for Windows (blast-2.2.17-ia32-win32.exe) against fasta formatted databases (UniProt or UniProt/Swiss-Prot). Information on signature/domain matches was obtained from the InterPro database (http:/www.ebi.ac.uk/InterPro). Pairwise local and global alignments of protein sequences were performed using programs of the Emboss package. HMMer analyses [65] were performed with the hmmer-2.3.2 software (hmmsearch, hmmalign; http://hmmer.wustl.edu/). Multiple protein sequence alignments were performed using either the hmmalign or Muscle v3.6 [66] software. Alignments were edited/trimmed where necessary using Jalview-1.7.5b [67]. Maximum likelihood trees were generated from the protein alignments using TreeBest v1.9.2 [68] along with the phylml option and the default WAG substitution model. Trees were displayed in circle format in TreeExplorer 2.12.

### Antibodies

A specific peptide (AERLHQENAARERR) from TbMyo1 was synthesized and coupled, via an added N-terminal cysteine residue, to a carrier protein (KLH) and used to immunize rabbits. At various intervals the immune response was determined by an ELISA using the peptide antigen. A peptide affinity resin was generated by attaching the peptide to Sulpho-Link resin (Pierce) via the N-terminal cysteine after treatment of the peptide with immobilized TCEP disulfide reducing gel (Pierce). The resin (approximately 1 ml) was placed into a column and washed with 10 volumes of column buffer (Tris-Cl 20 mM, NaCl 100 mM, pH 7.5). Rabbit immune serum was dialysed at 4°C overnight against column buffer and then cycled over the affinity resin in a closed loop at a flow rate of 0.2 ml overnight. The following day the column was washed with coupling buffer to remove nonspecific antibodies. Specifically bound antibodies were eluted by washing the resin with 10 column volumes of glycine (100 mM, pH 2.8) followed by 10 volumes of coupling buffer. Fractions (1.8 ml) were collected into tubes containing 0.2 ml of Tris-Cl (1 M, pH 7.5). Those fractions containing antibodies eluted from the resin at the low pH step were dialysed against Hepes buffer (10 mM, NaCl 150 mM, pH 7.5). The antibodies were stored at −80°C. Monoclonal IgM antibodies (CB1) against trypanosome lysosomal membrane glycoprotein p67 were obtained from Cedarlane Laboratories, Ontario, Canada.

### Cell Culture

Bloodstream cells (strain 328.114) were grown in HMI-9 media containing 10% fetal calf serum and G418 (2.5 µg.ml^−1^) [69]. Parental MITat 1.1 bloodstream cells were isolated from infected rats as described elsewhere [70]. Procyclic cells (strain 29.13) were grown in SDM-79 containing 10% fetal calf serum and G418 (25 µg.ml^−1^) and hygromycin (25 µg.ml^−1^) as described previously [71]. These cell lines contain a T7 polymerase and tetracycline repressor and were used for the conditional RNAi experiments.

### RNAi constructs

A 745 bp fragment was amplified using the following primers CCATCCTCGAGGAACTTCGCGACG and GCGTTAGCTTGGATCCACAGCATTCACAGG and cloned into the p2T7-177 RNAi vector using the restriction sites Xho I and BamH I (sites underlined). The construct was linearized with Not I and used to transfect cell lines using the Amaxa parasite nucleofection kit (Lonza). After phleomycin selection (2.5 µg.ml^−1^) expression of the dsRNA was induced by addition of tetracycline (1 µg.ml^−1^).

### Relative quantification of the *TbMyo1* transcript

The relative level of expression of the *TbMyo1* mRNA was estimated by quantitative reverse transcriptase PCR (QRT-PCR) using the Brilliant® SYBR Green qRT-PCR Master Mix Kit (1-step) from Stratagene in an MxPro 3000 instrument (Stratagene). The analysis was conducted using total RNA or mRNA fractions isolated using the Stratagene Absolutely RNA or Absolutely mRNA purification kits. Typically the reaction (25 ųl) contained total RNA (50 ng), the appropriate concentration of SYBR Green Master Mix (Stratagene) and forward and reverse primers. The primers were designed using Beacon Designer (Premier Biosoft) and for TbMyo1 were GCATCTGGACCTTAATAAAC (forward primer) and ATTAGGAATGTTCTTGAATGG (reverse primer) and for actin were ATGAGCAAGCGATGATGG (forward primer) and CAACTCGTTATAGAAGGTATGG (reverse primer). The assays were optimised for primer concentration, PCR reaction efficiency, precision, sensitivity and production of a single amplicon. The levels of TbMyo1 RNA were normalized against actin mRNA and the relative quantification was calculated by the ΔΔCt method and expressed as a ratio of induced to noninduced or of bloodstream to procyclic form as described previously [72].

### Immunoblotting

Western blots were performed as described previously [9]. Membranes were blocked in Tris buffer saline (Tris-Cl 25 mM, NaCl 150 mM, pH 7.5) containing 5% milk powder, 2.5% fetal calf serum and 0.5% Triton X-100. Affinity purified TbMyo1 antibodies and trypanosome anti-actin antibodies were used at dilutions of 1/250 and 1/1000 respectively in the blocking reagent.

### Immunofluorescence

Cells were purified from infected rat blood using DEAE chromatography, washed and resuspended (10^7^ cells.ml^−1^) in iso-osmotic PSG (pH 8.0) containing NaCl (44 mM), KCl (5 mM), Na_2_HPO_4_ (57 mM), NaH_2_PO_4_ (3 mM), glucose (10 mM), sucrose (70 mM). The cell suspension was mixed gently by inversion several times with an equal volume of freshly prepared paraformaldehyde (6%, w/v) in phosphate buffered saline (PBS) adjusted to pH 7.6. The suspension was incubated on ice for 10 min. After washing (600×g for 8 min at 4°C) in iso-osmotic PSG buffer the fixed cells were suspended at 2×10^7^ cells.ml^−1^ and applied to poly-lysine coated slides. After attachment of the cells the slides were washed with PBS containing glycine (10 mM) followed by another wash with PBS. The fixed cells were blocked by incubation in PBS containing BSA (1%), FCS (5%) and (0.25% Triton X-100) for 3 hours or overnight at 4°C. Primary antibodies were prepared in the blocking buffer. Incubation times were 1 hr at room temperature or overnight at 4°C. The slides were then washed with PBS and then incubated with Alexa-labelled secondary antibodies (Molecular Probes) typically diluted 1/1000 in the blocking buffer. The cells were incubated with the secondary antibodies for 1 h at 4°C, and then washed three times with PBS. The cells were mounted in Pro-Long Gold anti-fade reagent containing DAPI (Molecular Probes) and examined using a Zeiss Axiovert 100 fluorescence microscope or an Olympus Fluoview 1000 confocal microscope. For quantitative analysis of the colocalization data each optical section of a stack was analysed with the “Coloc” module of the Imaris image analysis software (IMARIS 7.0.0, Bitplane, Zurich, Switzerland). The results were expressed as a Pearson's correlation coefficient for voxel intensity correlation between the green (TbMyo1) and red (marker) channels. The intensity threshold in both channels was automatically determined using the algorithm in the “Coloc” software. Iso-surface rendering was performed on the combined Z-stack images using Imaris imaging software.

### Electron Microscopy

Cells were fixed in culture media with electron microscope grade glutaraldehyde (2.5%) for 30 min at room temperature with frequent inversions. After washing with PBS the cells were suspended in PBS and then embedded in agarose (2%) and then stained with osmium tetroxide (2%). The cells were then dehydrated in increasing concentrations of ethanol series and embedded in epoxy resin (Agar Scientific). Polymerization of the resin was performed at 60°C for 24 h. Sections were cut with an ultra-microtome and cells were contrasted with 0.5% uranyl acetate and Reynold's lead citrate. The sections were examined on a Jeol 2100 transmission electron microscope at 100 kV.

### Endocytosis assays

Endocytosis assays were performed using Texas-red labelled tomato lectin (20 ųg.ml^−1^) (Vector Laboratories) essential as described previously [41]. Samples were removed at various time points and immediately diluted 10-fold into ice cold iso-osmotic PSG cells, washed at 4°C, resuspended in ice cold PSG and immediately fixed with paraformaldehyde (final 3%). The cells were fixed on ice for 30 min, washed with PBS and resuspended at 1×10^7^ cells.ml^−1^ and applied to poly-lysine coated slides. The cells were mounted in Pro-Long Gold anti-fade reagent containing DAPI (Molecular Probes) and examined using an Olympus Fluoview 1000 confocal microscope or a Zeiss Axiovert 100 fluorescence microscope.

## Supporting Information

Figure S1 Maximum likelihood phylogenetic tree constructed using HMMALIGN and PTHR13140, showing classification of T. brucei myosins: Q381F5 and Q585L2 A set of 235 reviewed (curated) protein entries was obtained from the Swiss-Prot section of UniProt, each having at least one match to the Panther HMM model PTHR13140. Q381F5_9TRYP (identical to Tb11.01.7990) and Q585L2_9TRYP (identical to Tb927.4.3380) were added to this set, and the 237 proteins were aligned to the PTHR13140 HMM, which covers the myosin head domains, using HMMALIGN. The resulting alignment was edited in JALVIEW to remove N and C-terminal sequences not matching the model. The resulting alignment was 9133 in length. Residues to the right (C-terminal) of 2921 and to the left (N-terminal) of 1222 were trimmed, to give a final alignment of length 1292. At this stage, sequences with excessively short stretches of residues matching the model (including fragments) were removed. The final alignment contained 212 sequences including the query T. brucei myosins. The trimmed alignment was subjected to tree building using TREEBEST with the phyml option and the default WAG substitution model, to give a maximum likelihood tree. The tree was displayed using TREEEXPLORER as a circle tree. T. brucei myosins are shown marked with a filled triangle.
(0.03 MB PDF)

Figure S2 Maximum likelihood phylogenetic tree constructed by re-aligning, using MUSCLE, the trimmed protein sequences obtained from the HMM alignment shown in Figure S1 The trimmed alignment used for Figure S1, which covers the PTHR13140 matching region containing the Myosin head domains, was re-aligned using Muscle. The resulting alignment was subjected to tree building using TREEBEST with the phyml option and the default WAG substitution model, to give a maximum likelihood tree. The tree was displayed using TREEXPLORER as a circle tree. T. brucei myosins are shown marked with a filled triangle.
(0.03 MB PDF)

Figure S3 Maximum likelihood phylogenetic tree constructed by aligning full length protein sequences using MUSCLE The set of 237 proteins used in Figures S1 was aligned using MUSCLE. A maximum likelihood tree was constructed as described for Figures S1 and S2. T. brucei myosins are shown marked with a filled triangle.
(0.03 MB PDF)

Figure S4 Alignment of the N-terminal head or motor domain of class I myosins including TbMyo1/Q585L2 The larger alignment of 212 myosins to the PTHR13140 HMM for the N-terminal myosin motor domain was pruned using T-COFFEE to retain only the 41 class I myosins, including Q585L2, which segregated together in the same clade of the resulting phylogenetic tree shown (see Fig. S1). The MYOK_DICDI protein was subsequently removed to facilitate the display of the pruned alignment (as it contained long insertions in the head domain). The resulting alignment was displayed printed using JALVIEW using the clustalx color scheme. The conserved ATP-binding, actin-binding and IQ motif regions are annotated on the alignment, as indicated in the feature annotation of the UniProt entries. It should be noted that Q585L2 did not match the InterPro signature for the IQ calmodulin-binding motif (IPR000048) found in other myosins (see Table 2). However, the presence of a single IQ motif was found, in accordance with Foth et al. (2006) [14], consisting of IQ[RK]xxRxxxxx[RK].
(0.31 MB PDF)

Figure S5 Alignment of the C-terminal sequences of class I myosins including TbMyo1/Q585L2 A partial alignment of the myosin sequences including TbMyo1 (see Figure S4) was manually constructed from two separate alignments. First, full length sequences were aligned to PF06017 (Myosin_TH1/IPR010926) and, secondly, to PF00018 (SH3/IPR001452) HMM models using HMMALIGN of HMMER2. Only sequence regions matching the domain HMMs were aligned; therefore, the alignment includes some unaligned regions which are indicated. Unaligned N-terminal sequences including the head or motor domain and IQ motif(s) were trimmed off using JALVIEW. The alignment is presented using the clustalx color scheme. In the case of TbMyo1, the alignment shows: (1), the unaligned WW domain at positions 786 to 817 (which is missing in the other class I myosins). (2), the presence of a TH1 domain which lacks the N-terminal 18 residues of the domain and is interrupted by the insertion of a putative FYVE domain following the conserved lysine (at position 210 in the alignment). This insertion occurs roughly in the middle of the TH1 domain between positions 932/933 in TbMyo1. For the purpose of clarity, we do not show the remainder of the TbMyo1 sequence C-terminal of position 932, containing the FYVE domain sequence and the remaining C-terminal portion of the TH1 domain (992–1080). However, the alignment of the remaining C-terminal portion of the TbMyo1 TH1 domain was confirmed using BLASTP and the InterPro data for PF06017 (see Table 2). (3), the absence of the TH2, SH3 and TH3 domains, which are replaced by additional C-terminal sequence (at positions 1081–1167). This sequence C-terminal of the TH1 sequence in TbMyo1 was found to be unrelated to the TH3 acidic domain present in most of other class I myosins, as confirmed by an independent alignment of these regions (not shown). For reference, the entire sequence of TbMyo1 is shown underneath the alignment showing the WW domain (red), the interrupted TH1 domain (green) and the putative FYVE domain (purple).
(0.25 MB PDF)

Figure S6 Comparison of the localization of TbMyo1 and actin in bloodstream forms of T. brucei. Separate immunolocalizations were performed on the same population of fixed bloodstream forms using anti-actin or anti-TbMyo1 antibodies. The cells were visualized using a Zeiss Axiovert 100 fluorescence microscope and processed using Axiovision software. The images present the localization of actin (red) and TbMyo1 (green). Bar  = 5 µm
(0.91 MB EPS)

Figure S7 Colocalization of TbMyo1 with internalized tomato lectin in bloodstream forms of T. brucei. Texas red labelled tomato lectin was used as a surrogate cargo in the endocytosis assay performed at 37°C or 0°C as described in the methods. At various times the assay was cold stopped, the cells were fixed and immunolocalizations were performed with anti-Tbmyo1 antibodies. The images present the localization of Tomato lectin (red) and TbMyo1 (green). The nucleus and kinetoplast are also shown (blue). Bar  = 5 µm
(2.94 MB EPS)

Table S1 Identity mapping to UniProt and other descriptive information on myosins annotated in the Trypanosoma brucei proteome Identity mapping to UniProt was performed using blastp and global alignment as follows: (a) Tb11.01.7990 Blastp of Tb11.01.7990 against UniProt gave the following top hit. Q381F5_9TRYP  = > Score  = 2144 bits (5555), Expect  = 0.0, Method: Composition-based stats. Identities  = 1059/1059 (100%), Positives  = 1059/1059 (100%) UniProt Description for Q381F5_9TRYP: SubName: Full = Myosin, putative; (Trypanosoma brucei) (b) Tb927.4.3380 Blastp of Tb927.4.3380 against UniProt gave the following top hit. Q585L2_9TRYP  = > Score  = 2271 bits (5885), Expect  = 0.0, Method: Composition-based stats. Identities  = 1167/1167 (100%), Positives  = 1167/1167 (100%) UniProt Description for Q585L2_9TRYP: SubName: Full = Myosin IB heavy chain, putative; (Trypanosoma brucei) These identities were confirmed using Needleman-Wunsch global alignments.
(0.05 MB PDF)

Table S2 Information on InterPro signature matches for Q585L2 (TbMyo1) Data for signature matches with true status was obtained from InterPro. Matches are ordered N to C-terminal. Over the first N-terminal 775 amino acids, there are several matches with PFAM, SMART and PRINTS signatures, corresponding to the Myosin head, motor domain (InterPro Entry IPR001609). Hits for WW, FYVE and Myosin tail domains were observed in the remaining C-terminal sequence of the protein. It should be noted, the PFAM signature PF06017 (the sole signature of IPR010926) was originally annotated as Myosin_tail_2, but has been renamed to Myosin_TH1. However, IPR010926 is still confusingly named Myosin_tail_2. The alternative PFAM myosin tail domain signature, PF01576 (Myosin_tail_1), which is contained in IPR002928 (Myosin_tail), does not match this protein. See Figure 1 for a summary of the TbMyo1 domain structure based on the matches.
(0.03 MB PDF)

Table S3 Properties of various FYVE HMM matches against Q585L2 (TbMyo1) and related UniProt myosins. HMM models for various FYVE or FYVE/PHD domains were downloaded and run using HMMSEARCH (HMMER2.0) against a set of 1,700 Myosin proteins obtained from UniProt (defined as the set of all non-fragment proteins with hits to PTHR13140 - which covers the Myosin head region). The parent InterPro Entry associated with each HMM is shown in parentheses. Rank (in bold; rank 1 =  top hit), bit scores, and e-values, respectively, were recorded for all HMM hits. Percentage identity (PID) of Q585L2 to each of the 1699 other proteins was determined using the EMBOSS needle (global) and water (local) programs, and values including rank were recorded. Note: The superfamily HMM model - SSF57903 targets both the FYVE and PHD domains. However, only those individual models directed against the FYVE domain were used; models targeting the composite FYVE/PhD domain (0037409, 0037717, 0041001, and 0041002 in SSF57903) did not give any hits to Q585L2 and related myosins.
(0.02 MB PDF)

Table S4 E-values and other properties of the Myosins with potential hits to FYVE domain HMMs Of the 1600 proteins in the composite set of UniProt myosins and FYVE domain containing proteins, with hits to the downloaded HMMs, 31 were annotated as myosins. E-values for 4 of the HMMs are shown. None of the proteins shown are annotated as containing the FYVE domain. Q585L2 is shown in red.
(0.18 MB PDF)

Table S5 Ranking of the 19 T. brucei proteins with hits to the FYVE domain Q585L2 was ranked 18 out of 19 among the T. brucei proteins with hits to the SSF57903-0036632 HMM model (InterPro Entry IPR011011), showing that it is a relatively weak hit. The HMMs were run using HMMSEARCH (HMMER2.0). Q585L2 (Tb-Myo1) is shown in red. Data was generated using a bash shell script as follows: for ac in ′gg '⩓OS Trypanosoma brucei' sptr.hmmsearch.SSF57903-0036632.hmm.sptr.hybrib.FYVE-myosin-set | seq -topic = CCSI | awk '{print $1}' | egrep -vi 'belongs|contains'′; do cat -n acs.hmmsearch.SSF57903-0036632.hmm.sptr.hybrib.FYVE-myosin-set | grep $ac; done | sort
(0.10 MB PDF)

Table S6 Properties of FYVE domain HMM matches against kinetoplastid myosins and other FYVE domain-containing proteins in UniProt. A hybrid set of proteins was constructed (3,238 proteins in total), consisting of (i) the 1700 UniProt myosins with hits to PTHR13140, plus (ii) the FYVE domain-containing proteins from UniProt, as defined by all hits to InterPro Entries IPR000306, IPR011011 and IPR017455. The HMMs were run using HMMSEARCH (HMMER2.0). Relative rank (in bold, rank 1 =  top hit), bit scores and e-values, respectively, were recorded for all HMM hits in this hybrid set. Number of domain hits, were also recorded. A total of 1600 proteins in this set hit at least one of the FYVE domain HMM models. Protein hits selected for display have varying ranks and include myosins Q585L2 (Tb-Myo1) and related Q4Q3A5 (Lm-Myo1) and non-myosin proteins annotated by curators to contain the FYVE domain. The parent InterPro Entry associated with each HMM is shown in parentheses.
(0.03 MB PDF)
