# Supplementary figures and images for: Identification and Characterization of an Unusual Class I Myosin Involved in Vesicle Traffic in Trypanosoma brucei
Source: PLoS One. 2010 Aug 19;5(8):e12282. doi: 10.1371/journal.pone.0012282 (PMC2924389; doi:10.1371/journal.pone.0012282)

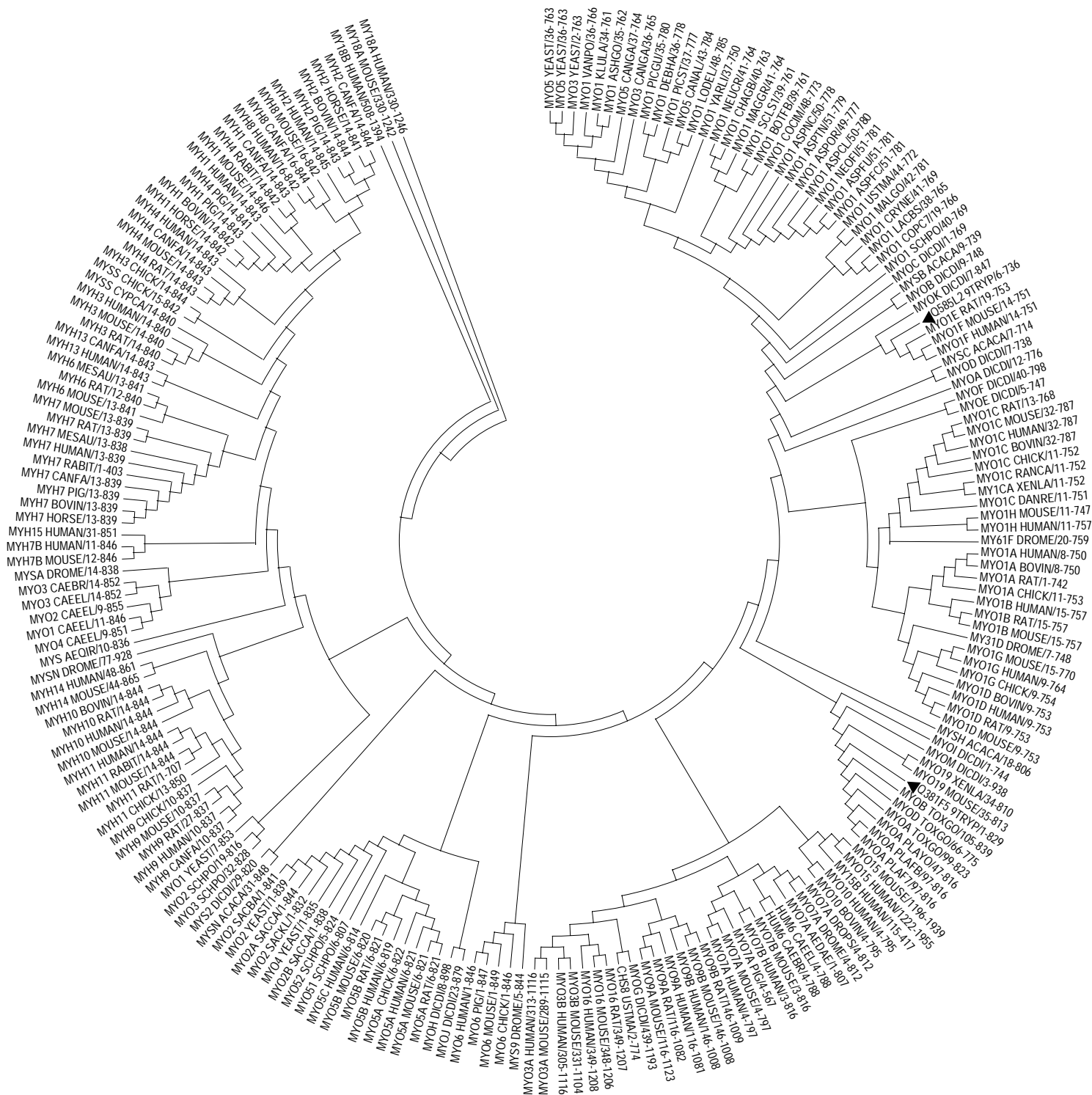

Supplement: Figure S1 — Maximum likelihood phylogenetic tree constructed using HMMALIGN and PTHR13140, showing classification of T. brucei myosins: Q381F5 and Q585L2 A set of 235 reviewed (curated) protein entries was obtained from the Swiss-Prot section of UniProt, each having at least one match to the Panther HMM model PTHR13140. Q381F5_9TRYP (identical to Tb11.01.7990) and Q585L2_9TRYP (identical to Tb927.4.3380) were added to this set, and the 237 proteins were aligned to the PTHR13140 HMM, which covers the myosin head domains, using HMMALIGN. The resulting alignment was edited in JALVIEW to remove N and C-terminal sequences not matching the model. The resulting alignment was 9133 in length. Residues to the right (C-terminal) of 2921 and to the left (N-terminal) of 1222 were trimmed, to give a final alignment of length 1292. At this stage, sequences with excessively short stretches of residues matching the model (including fragments) were removed. The final alignment contained 212 sequences including the query T. brucei myosins. The trimmed alignment was subjected to tree building using TREEBEST with the phyml option and the default WAG substitution model, to give a maximum likelihood tree. The tree was displayed using TREEEXPLORER as a circle tree. T. brucei myosins are shown marked with a filled triangle. (0.03 MB PDF) [file pone.0012282.s001.pdf]

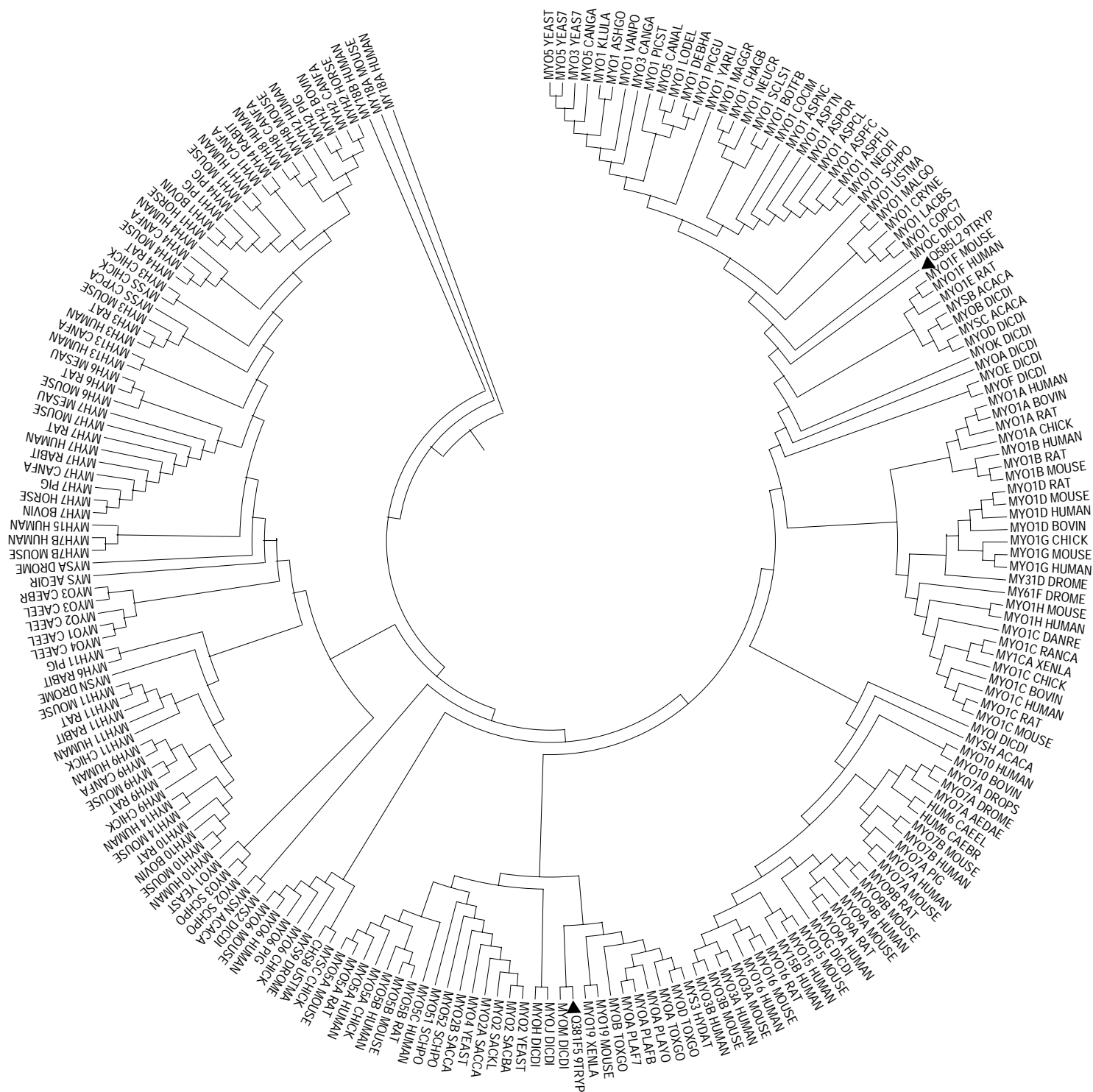

Supplement: Figure S2 — Maximum likelihood phylogenetic tree constructed by re-aligning, using MUSCLE, the trimmed protein sequences obtained from the HMM alignment shown in Figure S1 The trimmed alignment used for Figure S1, which covers the PTHR13140 matching region containing the Myosin head domains, was re-aligned using Muscle. The resulting alignment was subjected to tree building using TREEBEST with the phyml option and the default WAG substitution model, to give a maximum likelihood tree. The tree was displayed using TREEXPLORER as a circle tree. T. brucei myosins are shown marked with a filled triangle. (0.03 MB PDF) [file pone.0012282.s002.pdf]

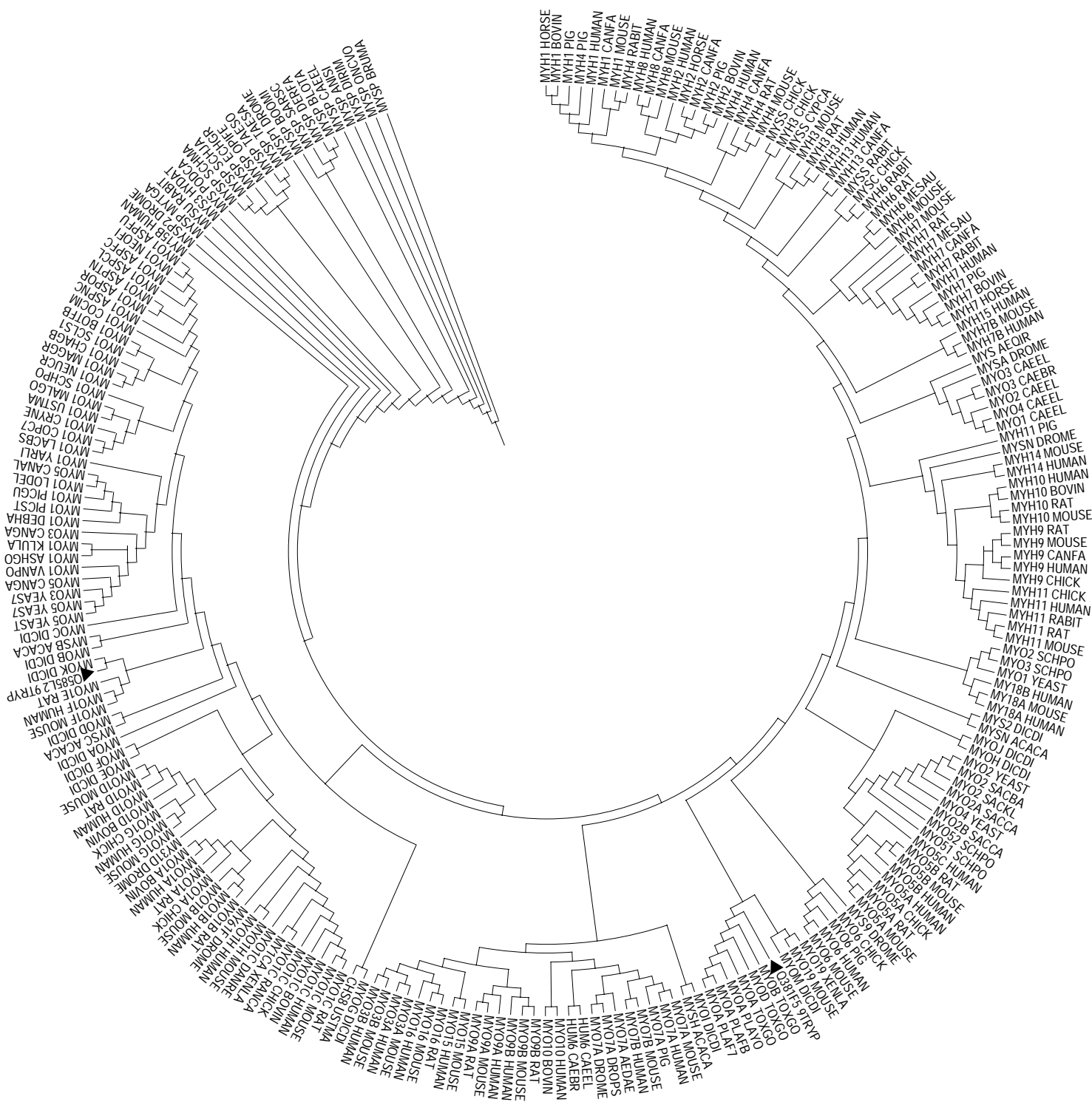

Supplement: Figure S3 — Maximum likelihood phylogenetic tree constructed by aligning full length protein sequences using MUSCLE The set of 237 proteins used in Figures S1 was aligned using MUSCLE. A maximum likelihood tree was constructed as described for Figures S1 and S2. T. brucei myosins are shown marked with a filled triangle. (0.03 MB PDF) [file pone.0012282.s003.pdf]

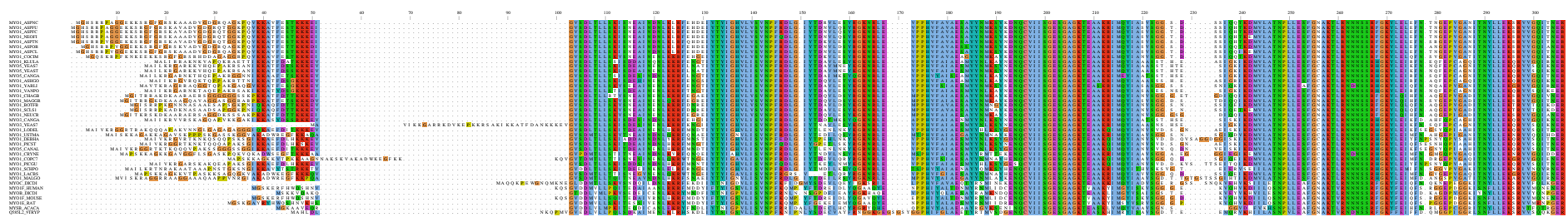

↑↑  
ATP-binding region

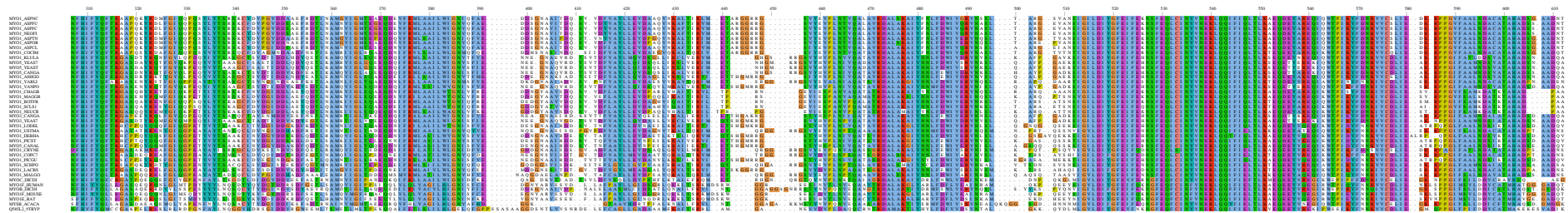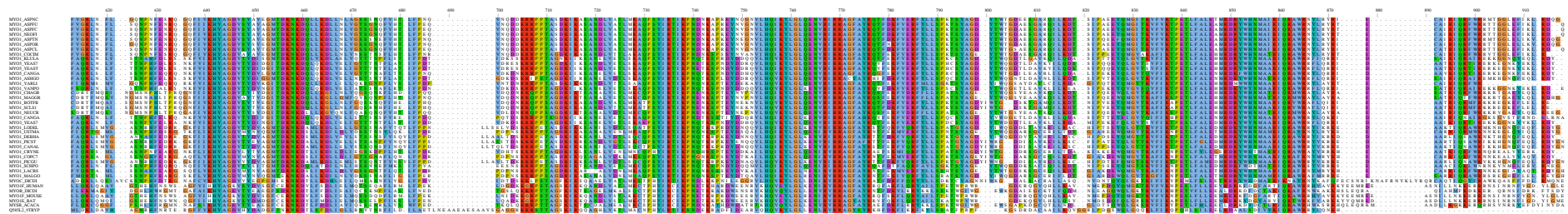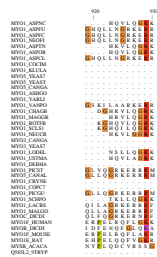

↑↑  
Actin-binding region

↑↑  
IQ motif

Supplement: Figure S4 — Alignment of the N-terminal head or motor domain of class I myosins including TbMyo1/Q585L2 The larger alignment of 212 myosins to the PTHR13140 HMM for the N-terminal myosin motor domain was pruned using T-COFFEE to retain only the 41 class I myosins, including Q585L2, which segregated together in the same clade of the resulting phylogenetic tree shown (see Fig. S1). The MYOK_DICDI protein was subsequently removed to facilitate the display of the pruned alignment (as it contained long insertions in the head domain). The resulting alignment was displayed printed using JALVIEW using the clustalx color scheme. The conserved ATP-binding, actin-binding and IQ motif regions are annotated on the alignment, as indicated in the feature annotation of the UniProt entries. It should be noted that Q585L2 did not match the InterPro signature for the IQ calmodulin-binding motif (IPR000048) found in other myosins (see Table 2). However, the presence of a single IQ motif was found, in accordance with Foth et al. (2006) [14], consisting of IQ[RK]xxRxxxxx[RK]. (0.31 MB PDF) [file pone.0012282.s004.pdf]
