# Supplementary material for: Identification and Characterization of an Unusual Class I Myosin Involved in Vesicle Traffic in Trypanosoma brucei
Source: PLoS One. 2010 Aug 19;5(8):e12282. doi: 10.1371/journal.pone.0012282 (PMC2924389; doi:10.1371/journal.pone.0012282)
Supplement: Figure S5 — Alignment of the C-terminal sequences of class I myosins including TbMyo1/Q585L2 A partial alignment of the myosin sequences including TbMyo1 (see Figure S4) was manually constructed from two separate alignments. First, full length sequences were aligned to PF06017 (Myosin_TH1/IPR010926) and, secondly, to PF00018 (SH3/IPR001452) HMM models using HMMALIGN of HMMER2. Only sequence regions matching the domain HMMs were aligned; therefore, the alignment includes some unaligned regions which are indicated. Unaligned N-terminal sequences including the head or motor domain and IQ motif(s) were trimmed off using JALVIEW. The alignment is presented using the clustalx color scheme. In the case of TbMyo1, the alignment shows: (1), the unaligned WW domain at positions 786 to 817 (which is missing in the other class I myosins). (2), the presence of a TH1 domain which lacks the N-terminal 18 residues of the domain and is interrupted by the insertion of a putative FYVE domain following the conserved lysine (at position 210 in the alignment). This insertion occurs roughly in the middle of the TH1 domain between positions 932/933 in TbMyo1. For the purpose of clarity, we do not show the remainder of the TbMyo1 sequence C-terminal of position 932, containing the FYVE domain sequence and the remaining C-terminal portion of the TH1 domain (992–1080). However, the alignment of the remaining C-terminal portion of the TbMyo1 TH1 domain was confirmed using BLASTP and the InterPro data for PF06017 (see Table 2). (3), the absence of the TH2, SH3 and TH3 domains, which are replaced by additional C-terminal sequence (at positions 1081–1167). This sequence C-terminal of the TH1 sequence in TbMyo1 was found to be unrelated to the TH3 acidic domain present in most of other class I myosins, as confirmed by an independent alignment of these regions (not shown). For reference, the entire sequence of TbMyo1 is shown underneath the alignment showing the WW domain (red), the interrupted TH1 domain (gre [file pone.0012282.s005.pdf]

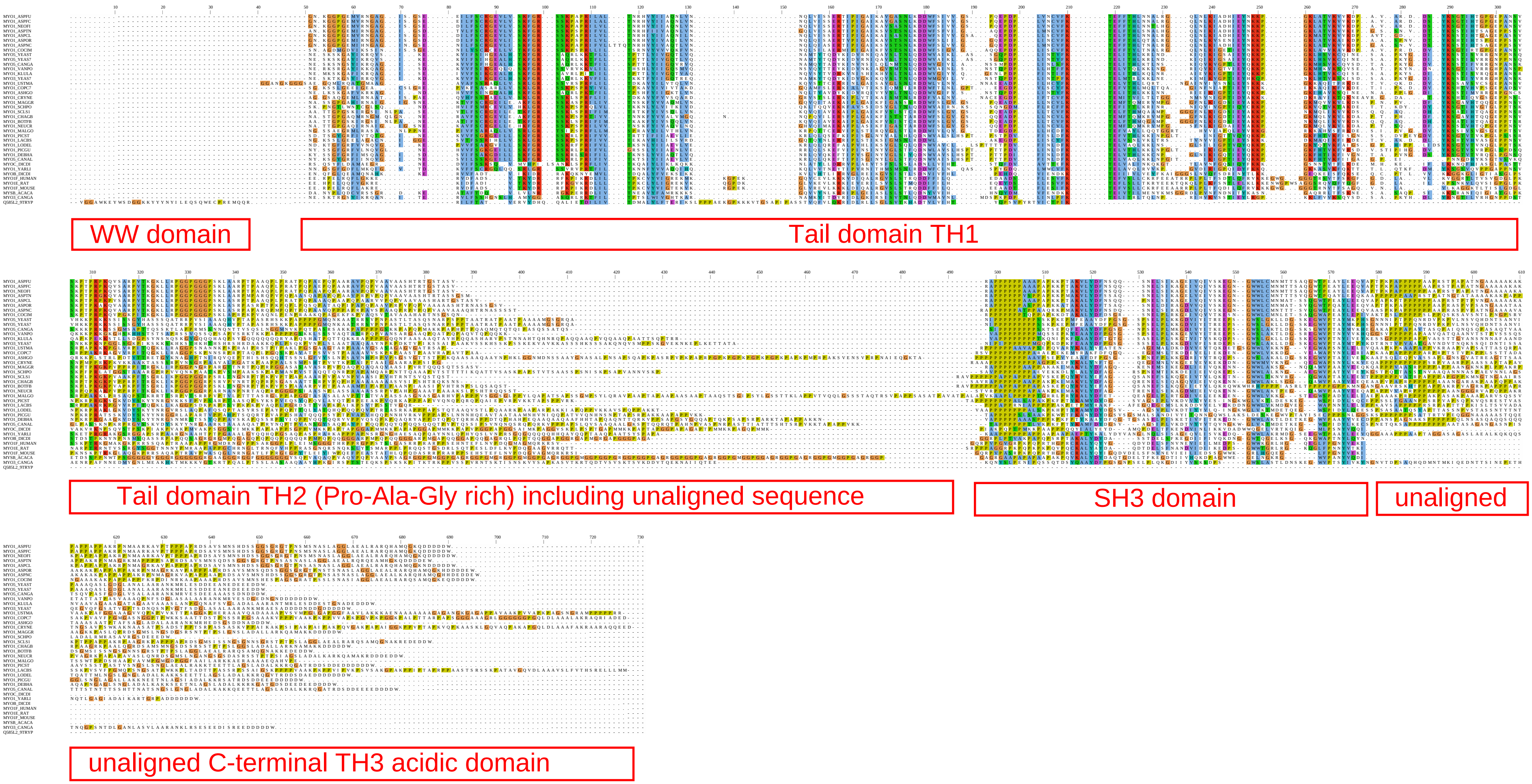
WW domain
Tail domain TH1
Tail domain TH2 (Pro-Ala-Gly rich) including unaligned sequence
SH3 domain
unaligned
unaligned C-terminal TH3 acidic domain
