# Supplementary material for: Identification and Characterization of an Unusual Class I Myosin Involved in Vesicle Traffic in Trypanosoma brucei
Source: PLoS One. 2010 Aug 19;5(8):e12282. doi: 10.1371/journal.pone.0012282 (PMC2924389; doi:10.1371/journal.pone.0012282)
Supplement: Table S1 — Identity mapping to UniProt and other descriptive information on myosins annotated in the Trypanosoma brucei proteome Identity mapping to UniProt was performed using blastp and global alignment as follows: (a) Tb11.01.7990 Blastp of Tb11.01.7990 against UniProt gave the following top hit. Q381F5_9TRYP = > Score = 2144 bits (5555), Expect = 0.0, Method: Composition-based stats. Identities = 1059/1059 (100%), Positives = 1059/1059 (100%) UniProt Description for Q381F5_9TRYP: SubName: Full = Myosin, putative; (Trypanosoma brucei) (b) Tb927.4.3380 Blastp of Tb927.4.3380 against UniProt gave the following top hit. Q585L2_9TRYP = > Score = 2271 bits (5885), Expect = 0.0, Method: Composition-based stats. Identities = 1167/1167 (100%), Positives = 1167/1167 (100%) UniProt Description for Q585L2_9TRYP: SubName: Full = Myosin IB heavy chain, putative; (Trypanosoma brucei) These identities were confirmed using Needleman-Wunsch global alignments. (0.05 MB PDF) [file pone.0012282.s008.pdf]

Systematic No.	UniProt Ac	Protein description	Size (residues)	Potential function	Signature	E value score	Residues
Tb11.01.7990	Q381F5	Myosin	1059	Motor protein	PTHR13140	0.0	1-1054
Tb927.4.3380	Q585L2	Myosin	1167	Motor protein	PTHR13140	0.0	11-775
