# Supplementary material for: Identification and Characterization of an Unusual Class I Myosin Involved in Vesicle Traffic in Trypanosoma brucei
Source: PLoS One. 2010 Aug 19;5(8):e12282. doi: 10.1371/journal.pone.0012282 (PMC2924389; doi:10.1371/journal.pone.0012282)
Supplement: Table S2 — Information on InterPro signature matches for Q585L2 (TbMyo1) Data for signature matches with true status was obtained from InterPro. Matches are ordered N to C-terminal. Over the first N-terminal 775 amino acids, there are several matches with PFAM, SMART and PRINTS signatures, corresponding to the Myosin head, motor domain (InterPro Entry IPR001609). Hits for WW, FYVE and Myosin tail domains were observed in the remaining C-terminal sequence of the protein. It should be noted, the PFAM signature PF06017 (the sole signature of IPR010926) was originally annotated as Myosin_tail_2, but has been renamed to Myosin_TH1. However, IPR010926 is still confusingly named Myosin_tail_2. The alternative PFAM myosin tail domain signature, PF01576 (Myosin_tail_1), which is contained in IPR002928 (Myosin_tail), does not match this protein. See Figure 1 for a summary of the TbMyo1 domain structure based on the matches. (0.03 MB PDF) [file pone.0012282.s009.pdf]

Method (Signature/Model)	Method name	InterPro Entry	Match Start Position	Match End Position	Method type	Match Date	Match E-value	Match Length	Comment
SSF52540	none	Unintegrated	6	744	HMM	02-MAY-2007	0	738	Myosin head, motor domain
SM00242	MYSc	IPR001609	6	714	HMM	17-AUG-2009	0	708	Myosin head, motor domain
G3DSA:3.30.538.10	none	Unintegrated	7	251	HMM	16-NOV-2006	2.3000E-76	244	Myosin head, motor domain
PTHR13140	MYOSIN	Unintegrated	11	775	HMM	27-APR-2007	0	764	Myosin head, motor domain
PTHR13140:SF31	MYOSIN I	Unintegrated	11	775	HMM	27-APR-2007	0	764	Myosin head, motor domain
PF00063	Myosin_head	IPR001609	14	700	HMM	30-OCT-2008	0	686	Myosin head, motor domain
PR00193	MYOSINHEAVY	IPR001609	42	61	Fingerprint	01-JUN-2009	6.8000E-50	19	Myosin head, motor domain
PR00193	MYOSINHEAVY	IPR001609	104	129	Fingerprint	01-JUN-2009	6.8000E-50	25	Myosin head, motor domain
PR00193	MYOSINHEAVY	IPR001609	150	177	Fingerprint	01-JUN-2009	6.8000E-50	27	Myosin head, motor domain
PR00193	MYOSINHEAVY	IPR001609	388	416	Fingerprint	01-JUN-2009	6.8000E-50	28	Myosin head, motor domain
PR00193	MYOSINHEAVY	IPR001609	441	469	Fingerprint	01-JUN-2009	6.8000E-50	28	Myosin head, motor domain
G3DSA:1.10.183.10	none	Unintegrated	267	380	HMM	16-NOV-	4.5000E-23	113	Myosin head,

						2006			motor domain
G3DSA:1.10.465.10	none	Unintegrated	405	649	HMM	16-NOV-2006	5.3000E-68	244	Myosin head, motor domain
SSF51045	WW_Rsp5_WWP	IPR001202	775	820	HMM	02-MAY-2007	8.2000E-09	45	WW domain
PS50020	WW_DOMAIN_2	IPR001202	784	817	Profile	24-AUG-2009	not available	33	WW domain
SM00456	WW	IPR001202	785	817	HMM	17-AUG-2009	.00000053	32	WW domain
PF00397	WW	IPR001202	786	815	HMM	30-OCT-2008	.000023	29	WW domain
PS01159	WW_DOMAIN_1	IPR001202	790	815	Pattern	24-AUG-2009	not applicable	25	WW domain
SSF57903	FYVE_PHD_ZnF	IPR011011	926	994	HMM	02-MAY-2007	.0000023	68	FYVE domain
PS50178	ZF_FYVE	IPR017455	931	993	Profile	24-AUG-2009	not available	62	FYVE domain
PF06017	Myosin_TH1	IPR010926	999	1074	HMM	30-OCT-2008	.0005	75	Myosin tail domain
