# Supplementary material for: Identification and Characterization of an Unusual Class I Myosin Involved in Vesicle Traffic in Trypanosoma brucei
Source: PLoS One. 2010 Aug 19;5(8):e12282. doi: 10.1371/journal.pone.0012282 (PMC2924389; doi:10.1371/journal.pone.0012282)
Supplement: Table S3 — Properties of various FYVE HMM matches against Q585L2 (TbMyo1) and related UniProt myosins. HMM models for various FYVE or FYVE/PHD domains were downloaded and run using HMMSEARCH (HMMER2.0) against a set of 1,700 Myosin proteins obtained from UniProt (defined as the set of all non-fragment proteins with hits to PTHR13140 - which covers the Myosin head region). The parent InterPro Entry associated with each HMM is shown in parentheses. Rank (in bold; rank 1 = top hit), bit scores, and e-values, respectively, were recorded for all HMM hits. Percentage identity (PID) of Q585L2 to each of the 1699 other proteins was determined using the EMBOSS needle (global) and water (local) programs, and values including rank were recorded. Note: The superfamily HMM model - SSF57903 targets both the FYVE and PHD domains. However, only those individual models directed against the FYVE domain were used; models targeting the composite FYVE/PhD domain (0037409, 0037717, 0041001, and 0041002 in SSF57903) did not give any hits to Q585L2 and related myosins. (0.02 MB PDF) [file pone.0012282.s010.pdf]

**Entry ID**	**UniProt Description**	**Species**	**Length**	**Rank and PID to Q585L2 (global;local)**	**SSF57903 0036632 (IPR011011)**	**SSF57903 0038788 (IPR011011)**	**SSF57903 0044739 (IPR011011)**	**SSF57903 0045116 (IPR011011)**	**PF01363.13 (ls) (IPR000306)**	**PF01363.13 (fs) (IPR000306)**	**SM00064 (IPR000306)**
Q4Q3A5_LEIMA	Myosin IB heavy chain, putative	Leishmania major	1373	**2**,48.4; **11**,48.4	**1**, 28.9, 1.7e-08	**1**, 25.6, 5.5e-09	**1**, 26.7, 1.1e-07	**2**, 15.6, 2e-05	**2**, 1.9, 5.7e-05	**2**, 6.5, 0.058	**2**, 4.6, 0.00017
A4I9R2_LEIIN	Myosin IB heavy chain, putative	Leishmania infantum	1372	**5**,47.6; **13**,47.7	**2**, 28.8, 1.9e-08	**2**, 25.4, 6.7e-09	**2**, 26.5, 1.3e-07	**3**, 14.5, 4.3e-05	**3**, 1.8, 5.9e-05	**3**, 6.5, 0.058	**1**, 4.6, 0.00017
Q4DAU2_TRYCR	Myosin IB heavy chain, putative	Trypanosoma cruzi	1165	**1**,69.1; **2**,69.3	**3**, 26.3, 9.6e-08	**4**, 21.6, 1.1e-07	**5**, 23.1, 1.1e-06	**1**, 22.5, 1.6e-07	**1**, 2.1, 5.5e-05	**1**, 10.6, 0.0038	**4**, -11.1, 0.0065
A4HAL3_LEIBR	Myosin IB heavy chain, putative	Leishmania braziliensis	1365	**3**,48; **12**,48	**4**, 25.9, 1.3e-07	**3**, 22.2, 6.8e-08	**4**, 23.7, 7.2e-07	**6**, 10.7, 0.00057	**5**, -6.3, 0.00047	**4**, 1.7 , 1.4	**3**, 1.3, 0.00037
Q585L2_9TRYP	Myosin IB heavy chain, putative	Trypanosoma brucei	1167	n/a	**5**, 21.2, 2.8e-06	**5**, 20.0, 3.6e-07	**3**, 23.9, 6.4e-07	**4**, 11.9, 0.00024	**4**, -2.2, 0.00016	**15**, -1.2, 9.5	**5**, -11.7, 0.0076
Q17D27_AEDAE	Myosin-rhogap protein, myr	Aedes aegypti	2258	**1221**,12.9; **503**,30.7	**6**, 6.7, 0.037	**6**, 7.0, 0.0057	**7**, 3.5, 0.21	**60**, -0.5, 1.4	**34**, -44.9, 9.7	no hit	**32**, -42.7, 9.8
