# Supplementary material for: Identification and Characterization of an Unusual Class I Myosin Involved in Vesicle Traffic in Trypanosoma brucei
Source: PLoS One. 2010 Aug 19;5(8):e12282. doi: 10.1371/journal.pone.0012282 (PMC2924389; doi:10.1371/journal.pone.0012282)
Supplement: Table S4 — E-values and other properties of the Myosins with potential hits to FYVE domain HMMs Of the 1600 proteins in the composite set of UniProt myosins and FYVE domain containing proteins, with hits to the downloaded HMMs, 31 were annotated as myosins. E-values for 4 of the HMMs are shown. None of the proteins shown are annotated as containing the FYVE domain. Q585L2 is shown in red. (0.18 MB PDF) [file pone.0012282.s011.pdf]

Accession	Protein length	Species	Curation status	UniProt description	SSF57903 0036632 (IPR011011)	SSF57903 0045116 (IPR011011)	PF01363.13 (ls) (IPR000306)	SM00064 (IPR000306)
Q4Q3A5	1373	Leishmania major	unreviewed	Myosin IB heavy chain, putative;	1.7e-08	3.8e-05	0.00011	0.00033
A4I9R2	1372	Leishmania infantum	unreviewed	Myosin IB heavy chain, putative;	1.9e-08	8.2e-05	0.00011	0.00033
C6JVZ1	1634	Phaeodactylum tricornutum	unreviewed	Myosin I;	3.2e-08	1.4e-08	0.00011	0.00011
Q4DAU2	1165	Trypanosoma cruzi	unreviewed	Myosin IB heavy chain, putative;	9.6e-08	3e-07	0.0001	0.012
A4HAL3	1365	Leishmania braziliensis	unreviewed	Myosin IB heavy chain, putative;	1.3e-07	0.0011	0.0009	0.00071
C6K3L9	1388	Crithidia deanei	unreviewed	Putative myosin IB heavy chain;	1.9e-07	5.8e-05	0.012	0.011
Q585L2	1167	Trypanosoma brucei	unreviewed	Myosin IB heavy chain, putative;	2.8e-06	0.00046	0.00031	0.014
Q17D27	2258	Aedes aegypti	unreviewed	Myosin-rhogap protein, myr;	0.037	2.6	no hit	no hit
A8PNG2	1988	Brugia malayi	unreviewed	Heavy chain, unconventional myosin protein 7, putative;	0.11	0.0009	7.6	1.2
B7Q880	1837	Ixodes scapularis	unreviewed	Myosin-IX, putative; EC=2.7.11.13;	1.2	0.48	3.4	3.4
Q9GZG6	1887	Caenorhabditis elegans	unreviewed	Heavy chain, unconventional myosin protein 7;	2.7	0.24	no hit	no hit
Q622K8	2099	Caenorhabditis briggsae	reviewed	Unconventional myosin heavy chain 6;	4.2	no hit	no hit	no hit
P91443	2098	Caenorhabditis elegans	reviewed	Unconventional myosin heavy chain 6;	4.2	no hit	no hit	no hit

Q8T6L7	1278	Drosophila melanogaster	unreviewed	Myosin 95E, isoform D; EC=3.6.1.3; SubName: Full=Unconventional myosin 95E isoform I	6.5	no hit	no hit	no hit
Q8T6L6	1258	Drosophila melanogaster	unreviewed	Myosin 95E, isoform B; EC=3.6.1.3; SubName: Full=Unconventional myosin 95E isoform II	6.5	no hit	no hit	no hit
Q4AB24	446	Drosophila melanogaster	unreviewed	Myosin 95E, isoform F; EC=3.6.1.3; SubName: Full=Myosin 95E, isoform G; EC=3.6.1.3	6.5	no hit	no hit	no hit
Q4AB27	1288	Drosophila melanogaster	unreviewed	Myosin 95E, isoform E; EC=3.6.1.3;	6.5	no hit	no hit	no hit
Q9V3Z6	2167	Drosophila melanogaster	reviewed	Myosin-VIIa; Short=DmVIIa; AltName: Full=Protein crinkled;	7.0	no hit	no hit	no hit
Q29P71	2168	Drosophila pseudoobscura	reviewed	Myosin-VIIa; AltName: Full=Protein crinkled;	7.0	no hit	no hit	no hit
C1M0R0	1513	Schistosoma mansoni	unreviewed	Myosin V, putative;	7.9	no hit	no hit	no hit
C1M0Q9	1832	Schistosoma mansoni	unreviewed	Myosin V, putative;	7.9	no hit	no hit	no hit
Q8C170	2542	Mus musculus	reviewed	Myosin-IXa; AltName: Full=Unconventional myosin-9a;	8.4	0.2	1.8	1.6
B2RTY4	2548	Homo sapiens	reviewed	Myosin-IXa; AltName: Full=Unconventional myosin-9a;	8.4	3.7	1.8	1.6
Q9Z1N3	2626	Rattus norvegicus	reviewed	Myosin-IXa; AltName: Full=Unconventional myosin-9a; AltName: Full=Myr 7;	8.8	0.28	1.8	1.6
Q17LW0	2163	Aedes aegypti	reviewed	Myosin-VIIa; AltName: Full=Protein crinkled;	9.6	no hit	no hit	no hit
B0WB21	2173	Culex quinquefasciatus	unreviewed	Myosin-VIIa;	9.6	no hit	no hit	no hit
A7ASN6	1473	Babesia bovis	unreviewed	Myosin B; EC=3.6.4.1;	10	no hit	no hit	no hit

Q68RJ2	1292	Danio rerio	unreviewed	Myosin VIa;	no hit	no hit	no hit	no hit
C4QLW6	1334	Schistosoma mansoni	unreviewed	Myosin xvIII, putative;	no hit	no hit	no hit	no hit
Q13459	2158	Homo sapiens	reviewed	Myosin-IXb; AltName: Full=Unconventional myosin-9b;	no hit	no hit	no hit	no hit
Q6B334	1253	Danio rerio	unreviewed	Myosin VIa;	no hit	no hit	no hit	no hit
