# Supplementary material for: Identification and Characterization of an Unusual Class I Myosin Involved in Vesicle Traffic in Trypanosoma brucei
Source: PLoS One. 2010 Aug 19;5(8):e12282. doi: 10.1371/journal.pone.0012282 (PMC2924389; doi:10.1371/journal.pone.0012282)
Supplement: Table S5 — Ranking of the 19 T. brucei proteins with hits to the FYVE domain Q585L2 was ranked 18 out of 19 among the T. brucei proteins with hits to the SSF57903-0036632 HMM model (InterPro Entry IPR011011), showing that it is a relatively weak hit. The HMMs were run using HMMSEARCH (HMMER2.0). Q585L2 (Tb-Myo1) is shown in red. Data was generated using a bash shell script as follows: for ac in ′gg '⩓OS Trypanosoma brucei' sptr.hmmsearch.SSF57903-0036632.hmm.sptr.hybrib.FYVE-myosin-set | seq -topic = CCSI | awk '{print $1}' | egrep -vi 'belongs|contains'′; do cat -n acs.hmmsearch.SSF57903-0036632.hmm.sptr.hybrib.FYVE-myosin-set | grep $ac; done | sort (0.10 MB PDF) [file pone.0012282.s012.pdf]

Count	Rank	Accession	UniProt Description
1	26	Q57U01	Putative uncharacterized protein
2	274	Q57V22	Zinc finger protein, putative
3	434	Q38DK7	Zinc finger protein, putative
4	616	Q38DV9	Putative uncharacterized protein
5	714	Q38F57	Protein kinase, putative
6	728	Q388P6	Putative uncharacterized protein
7	851	Q387A6	Phosphatidylinositol (3,5) kinase, putative
8	995	Q381I0	Zinc finger protein, putative
9	1199	Q57UC6	Putative uncharacterized protein
10	1209	Q57VR5	Zinc finger protein, putative
11	1223	Q387V2	Zinc finger protein, putative
12	1291	Q381Q2	Phosphatidylinositol 3-kinase catalytic subunit, putative
13	1366	Q38FZ0	Protein kinase, putative
14	1398	Q38AR4	Putative uncharacterized protein
15	1412	Q384A2	Protein kinase
16	1413	Q965D1	Zinc finger protein kinase
17	1419	Q4GYP3	Zinc-binding phosphatase, putative
18	1425	Q585L2	Myosin IB heavy chain, putative
19	1528	Q387R7	Class 3 lipase, putative
