# Supplementary material for: Identification and Characterization of an Unusual Class I Myosin Involved in Vesicle Traffic in Trypanosoma brucei
Source: PLoS One. 2010 Aug 19;5(8):e12282. doi: 10.1371/journal.pone.0012282 (PMC2924389; doi:10.1371/journal.pone.0012282)
Supplement: Table S6 — Properties of FYVE domain HMM matches against kinetoplastid myosins and other FYVE domain-containing proteins in UniProt. A hybrid set of proteins was constructed (3,238 proteins in total), consisting of (i) the 1700 UniProt myosins with hits to PTHR13140, plus (ii) the FYVE domain-containing proteins from UniProt, as defined by all hits to InterPro Entries IPR000306, IPR011011 and IPR017455. The HMMs were run using HMMSEARCH (HMMER2.0). Relative rank (in bold, rank 1 = top hit), bit scores and e-values, respectively, were recorded for all HMM hits in this hybrid set. Number of domain hits, were also recorded. A total of 1600 proteins in this set hit at least one of the FYVE domain HMM models. Protein hits selected for display have varying ranks and include myosins Q585L2 (Tb-Myo1) and related Q4Q3A5 (Lm-Myo1) and non-myosin proteins annotated by curators to contain the FYVE domain. The parent InterPro Entry associated with each HMM is shown in parentheses. (0.03 MB PDF) [file pone.0012282.s013.pdf]

Entry ID	UniProt Description/ Similarity comment	Species	Length	Number of FYVE domain hits	SSF57903 0036632 (IPR011011)	SSF57903 0038788 (IPR011011)	SSF57903 0044739 (IPR011011)	SSF57903 0045116 (IPR011011)	PF01363.13 (ls) (IPR000306)	PF01363.13 (fs) (IPR000306)	SM00064 (IPR000306)
B0WS48	RUN and FYVE domain-containing protein 1	Culex quinquefasciatus	507	3	**1**, 222.5, 3.4e-64	**1**, 204.2, 2.9e-66	**1**, 215.0, 6.2e-62	**1**, 194.2, 7e-59	**1**, 343.7, 1.1e-100	**1**, 338.0, 5.7e-99	**1**, 298.5, 4.4e-87
Q9HBF4	Zinc finger FYVE domain-containing protein 1/ Contains 2 FYVE-type zinc fingers	Homo sapiens	777	2	**8**, 127.5, 2.1e-37	**9**, 117.7, 2.2e-38	**8**, 114.3, 1.2e-31	**3**, 153.3, 1.4e-46	**6**, 168.9, 4.5e-48	**6**, 165.2, 1.6e-47	**127**, 114.4, 1.2e-31
Q69ZL1	FYVE, RhoGEF and PH domain-containing protein 6	Mus musculus	1399	2	**111**, 73.4, 3.7e-21	**114**, 68.9, 1.2e-22	**111**, 70.5, 2.9e-19	**277**, 61.7, 4.9e-19	**30**, 121.8, 7.1e-34	**32**, 119.9, 1.9e-34	**207**, 108.2, 8.8e-30
O14964	Hepatocyte growth factor-regulated tyrosine kinase substrate/ Contains 1 FYVE-type zinc finger	Homo sapiens	777	1	**116**, 75.1, 1.3e-21	**424**, 60.9, 4.4e-20	**72**, 72.9, 6.4e-20	**267**, 62.0, 4e-19	**237**, 108.2, 8.6e-30	**245**, 106.4, 1.6e-30	**29**, 120.2, 2.1e-33
Q4Q3A5	Myosin IB heavy chain, putative	Leishmania major	1373	1	**1359**, 28.9, 1.7e-08	**1383**, 25.6, 1.1e-08	**1359**, 26.7, 2.1e-07	**1503**, 15.6, 3.8e-05	**1387**, 1.9, 0.00011	**140**, 6.5, 0.11	**1384**, 4.6, 0.00033
Q7LKZ7	Beige protein homolog 1/ Contains 1 FYVE-type zinc finger	Schizosaccharomyces pombe	2609	1	**1390**, 26.7, 7.3e-08	**1358**, 28.7, 1.1e-09	**1390**, 22.5, 2.9e-06	**1328**, 30.4, 1.3e-09	**1370**, 8.4, 2e-05	**1407**, 6.1, 0.14	**1351**, 15.2, 2.8e-05
Q585L2	Myosin IB heavy chain, putative	Trypanosoma brucei	1167	1	**1425**, 21.2, 2.8e-06	**1425**, 20.0, 6.8e-07	**1377**, 23.9, 1.2e-06	**1515**, 11.9, 0.00046	**1394**, -2.2, 0.00031	no hit	**1422**, -11.7, 0.014
Q22366	Rab-3-interacting molecule unc-10/ Contains 1 FYVE-type zinc finger	Caenorhabditis elegans	1563	1	**1521**, 10.5, 0.0031	**1516**, 8.9, 0.0026	**1516**, 5.2, 0.14	**90**, 83.3, 1.5e-25	no hit	no hit	**1494**, -28.6, 0.71
